# Boron-doped nanographene: Lewis acidity, redox properties, and battery electrode performance†
†Electronic supplementary information (ESI) available: Supporting movie, experimental procedure and characterization details. CCDC 1059942–1059945. For ESI and crystallographic data in CIF or other electronic format see DOI: 10.1039/c5sc02246k


**DOI:** 10.1039/c5sc02246k

**Published:** 2015-09-24

**Authors:** Shinichiro Osumi, Shohei Saito, Chuandong Dou, Kyohei Matsuo, Keita Kume, Hirofumi Yoshikawa, Kunio Awaga, Shigehiro Yamaguchi

**Affiliations:** a Department of Chemistry , Graduate School of Science , Nagoya University , Furo, Chikusa , Nagoya 464-8602 , Japan . Email: s_saito@chem.nagoya-u.ac.jp; b Institute of Transformative Bio-Molecules (WPI-ITbM) , Nagoya University , Furo, Chikusa , Nagoya 464-8602 , Japan . Email: yamaguchi@chem.nagoya-u.ac.jp; c CREST , Japan Science and Technology Agency (JST) , Furo, Chikusa , Nagoya 464-8602 , Japan

## Abstract

The preparation of boron-doped nanocarbon scaffolds with well-defined structures is important for the understanding of the impact of boron doping on their properties and behavior at the molecular level. We recently succeeded in the synthesis of a structurally well-defined nanographene molecule, bearing two boron atoms at the central positions. In this study, the characteristic properties and functions of this boron-doped nanographene were investigated in terms of (1) Lewis acidity, (2) redox properties, and (3) electrode performance in a battery. This boron-doped nanographene was susceptible to chemical adsorption with various Lewis bases, resulting in significant changes in the absorption and fluorescence properties, as well as in the conformation of the honeycomb framework. The two-electron reduction of this boron-doped nanographene produced a dianionic species that showed a substantial biradical character with a triplet ground state. A Li battery electrode, composed of a boron-doped nanographene with small peripheral substituents, displayed a stable performance in the 1.5–4.0 V range with a first discharge capacity of 160 mA h g^–1^. These results provide important insights into the effect of boron doping on nanocarbon compounds.

## Abstract 2

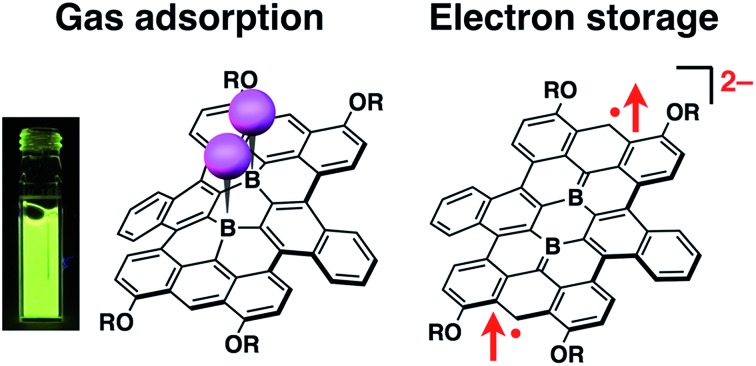
The impact of boron doping on the nature of nanographene was investigated at the molecular level in terms of chemical adsorption with various Lewis bases, spin multiplicity of the two electron-reduced species, and performance as a battery electrode.

## Introduction

Substitutional doping is a powerful method for the modulation of graphene properties.^1^ Replacement of individual carbon atoms with heteroatoms results in the disruption of the sp^2^-hybridized carbon network, giving rise to properties different from those of pristine graphene. Among a variety of possible dopants, boron has received particular attention, due to its unique characteristics including electron deficiency and Lewis acidity. Boron doping increases the performance of graphene-based devices, such as field effect transistors,^2^ Li-ion batteries,^3^ supercapacitors,^4^ and solar cells.^5^ However, despite intensive studies on the chemistry of boron-doped graphene, reliable methods for a controlled boron doping in terms of both doping site and density had not been fully established until structurally well-defined boron-doped graphene nanoribbon was recently reported by two independent groups.^6^ Conventional preparation methods, such as chemical vapor deposition,^7^ reductive coupling in solution,^8^ or thermal annealing,^9^ inevitably produce a mixture of boron-doped graphene compounds, which differ in size, edge structure, and doping pattern. In order to understand the impact of boron doping at the molecular level, the preparation of structurally well-defined boron-doped graphene is indispensible.

In order to obtain graphene systems with a uniform structure, a bottom-up synthesis from a defined organic precursor represents a promising approach.^10^ This technique is also effective for the synthesis of the doped graphene systems.^6^,^11^ So far, nitrogen-doped nanographene scaffolds with precise atomic structures have been successfully synthesized.^12^ The synthesis of boron-doped nanographene, on the other hand, still remains challenging due to the intrinsic instability of tri-coordinate organoboranes towards oxygen and moisture. One strategy to circumvent this obstacle is the incorporation of nitrogen atoms in the form of B–N units.^13^ Even though the chemical stability of the organoboranes is thus enhanced through the interaction between the lone pair of electrons on the nitrogen atom and the vacant p orbital of the boron atom, the intrinsic Lewis acidity of the boron atom is lost simultaneously.

**Scheme 1:**
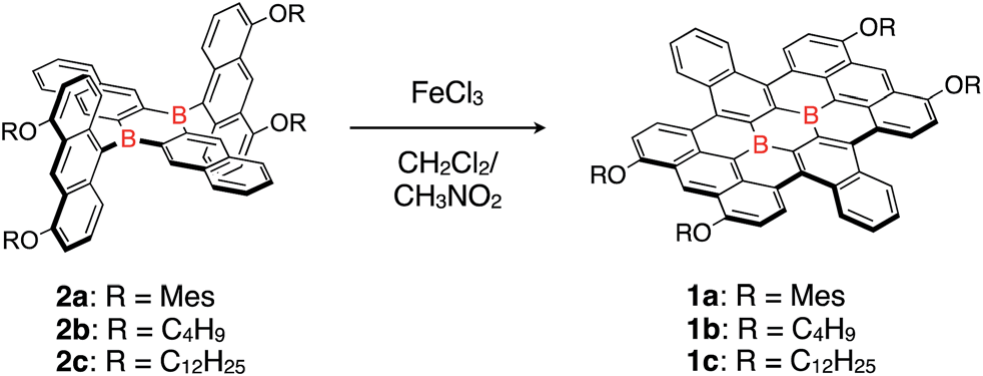
Synthesis of a honeycomb lattice framework, bearing two boron atoms at the central positions.

Recently, we introduced the concept of “structural constraint” as an alternative stabilization strategy for organoboranes.^14^ This design paradigm is based on the enforced planarization of triphenylboranes *via* methylene tethers, and resulted in great stability under ambient conditions.14*a* Using this strategy, a fully π-conjugated planar triarylborane, in which three aryl groups are directly fused with one another, was successfully produced.14*c* This success has also opened a new avenue for the precise synthesis of boron-doped nanographene scaffolds and enabled us to prepare a nanometer-sized honeycomb structure with two boron atoms at the central positions (Scheme 1).14*d*

Experimental and theoretical studies on boron-doped nanographene **1a** were able to reveal some of its characteristic properties arising from the presence of the two boron atoms, *e.g.* broad absorption bands in the visible region and reversible multi-redox processes. However, these findings describe merely parts of the chemistry of boron-doped nanographene. In this article, we disclose the following aspects of boron-doped nanographene: (1) Lewis acidity, (2) redox properties, and (3) electrode performance in batteries. These properties and functions are closely associated with the presence of the boron atoms and can thus be regarded as the impact of boron doping on pristine nanographene.

## Results and discussion

### Synthesis and structure

In the previously reported synthesis of boron-doped nanographene **1a**, bulky mesityloxy groups were introduced at the peripheral positions of the π-conjugated skeleton. This structural feature enabled the suppression of strong aggregation and gave rise to sufficient solubility in common organic solvents (Table 1). However, the less densely packed structure, arising from the presence of these bulky substituents, is unfavorable for attaining high levels of molecule-based device performance in *e.g.* organic field-effect transistors (OFET) and battery electrodes. Small orbital overlap causes low carrier mobility in OFET applications^15^ and weak intermolecular interaction brings about dissolution of the battery electrode materials into electrolyte, resulting in low cycle performance.^16^ This potential drawback can be overcome by the use of sterically less demanding substituents. In order to investigate such substituent effects on the packing structure and material properties, alkoxy-substituted derivatives **1b** and **1c** were synthesized and examined in this study.

**Table 1:** Solubility of boron-doped nanographenes at 25 °C

Solvent	**1a**	**1b**	**1c**
Chlorobenzene	4.8 mg mL^–1^	<1 mg mL^–1^	3.7 mg mL^–1^
*o*-Dichlorobenzene	10.8 mg mL^–1^	<1 mg mL^–1^	5.5 mg mL^–1^

Butoxy- and dodecyloxy-substituted boron-doped nanographenes **1b** and **1c** were prepared as described for **1a** (Scheme 1). The oxidative cyclodehydrogenation of dianthryl-substituted dihydrodiborapentacene precursors **2b** and **2c** using FeCl_3_ in a binary CH_2_Cl_2_/nitromethane solvent afforded **1b** and **1c** in 19% and 34% yield, respectively. Their yields were slightly lower than that of **1a** (51%),14*d* most likely due to their low solubility in common organic solvents, which required a laborious purification procedure. The introduction of the electron-donating aryloxy or alkoxy groups at the 4,5-positions of the terminal anthracene moiety is crucial for promoting the cyclization.^17^ Compounds **1a–1c** thus prepared are highly stable in air. Only subtle degradation was observed for **1a** and **1c** in toluene solutions in air even after 7 days by monitoring with the UV-visible absorption spectroscopy (Fig. S2†).

**Fig. 1:**
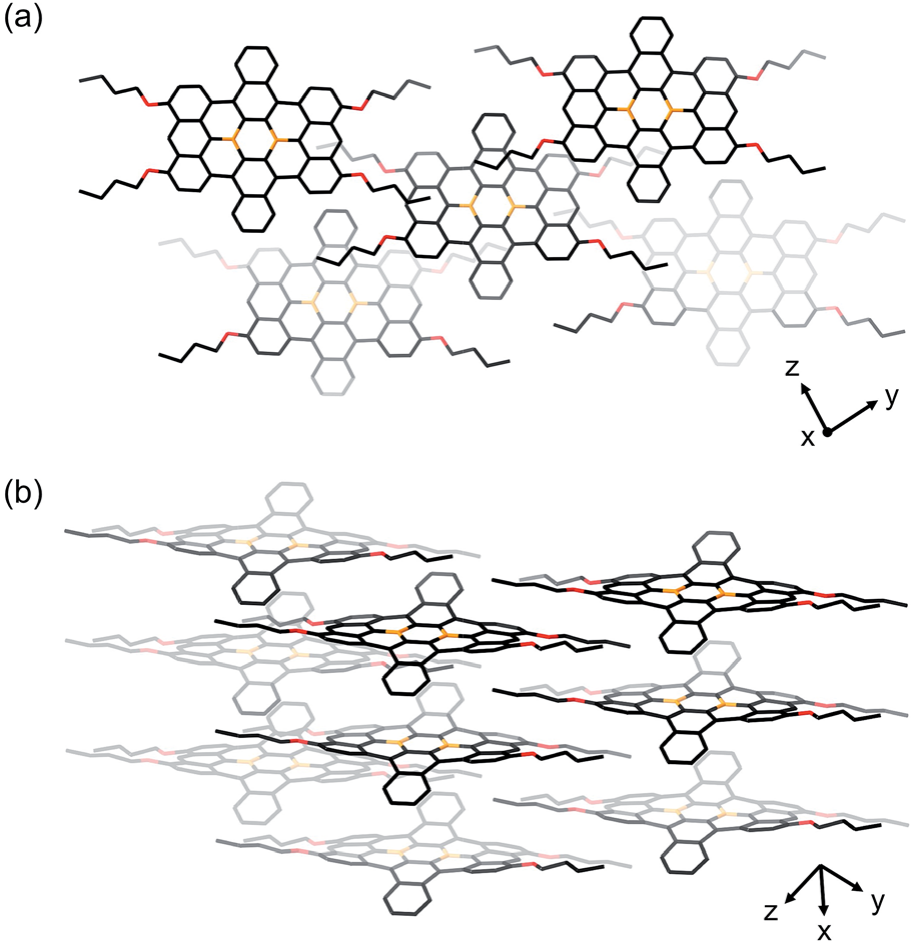
Crystal packing of **1b** from (a) a top view and (b) a side view. Hydrogen atoms are omitted for clarity. Orange: boron; red: oxygen.

Single crystals of **1b** and **1c** were obtained from 1,2-dichloroethane/2-propanol and chlorobenzene/octane solutions, respectively. X-ray crystallographic analyses confirmed that both compounds contain the four alkoxy groups at the zig-zag edges of the nanographene skeleton (Fig. 1 for **1b** and Fig. S20† for **1c**). The local BC_3_ sites adopt a planar geometry, in which the B–C bonds (1.50–1.54 Å) are much shorter than those of non-fused triphenylboranes (1.57–1.59 Å). The nanographene skeletons in both **1b** and **1c** are deviated from planarity due to the steric repulsion between the hydrogen atoms in the cove regions, and accordingly **1b** and **1c** do not form π-stacked structures. However, due to their sterically less demanding substituents, **1b** and **1c** are more densely packed than **1a**. This is consistent with the fact that **1b** and **1c** exhibit a significantly lower solubility than **1a** (Table 1). The use of 9,10-dihydro-9,10-diboraanthracene as a key precursor instead of **2** may potentially represent an option to generate an ideally planar fused skeleton and induce π-stacking. However, our previous study revealed that the treatment of this dihydrodiboraanthracene precursor with FeCl_3_ resulted in undesirable chlorination at the 1,8-position of the terminal anthracene moieties rather than cyclization.^18^ It should furthermore be noted that, in contrast to **1a**, no solvent molecule was contained in the crystal packing of **1b** and **1c**.

The photophysical properties of **1c** were investigated in CH_2_Cl_2_ (Fig. S1†). The absorption spectrum displayed a broad absorption band, which covered the entire visible region (400–700 nm) and contained a maximum at 563 nm. The fluorescence spectrum showed a broad emission band in the visible-NIR region with a maximum at 694 nm. These results are comparable to those of **1a**, and DFT calculations (B3LYP/6-31G*) supported the notion that the electronic structure of the nanographene scaffold remained unchanged upon substitution of the peripheral aryloxy groups with alkoxy groups (Fig. S22†).

### Lewis acidity

**Scheme 2:**
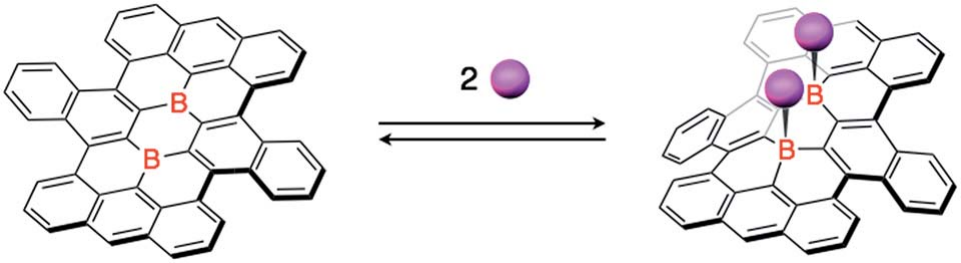
Chemical adsorption of Lewis bases on a boron-doped honeycomb lattice structure.

We previously demonstrated that planarized triarylboranes maintain Lewis acidity and are able to form coordination complexes with Lewis bases despite their polycyclic structure. Specifically, we revealed dynamic B–N bond formation processes in the presence of pyridine, which are responsible for the intriguing thermochromism14*c* and photodissociation14*e* observed for these B–N Lewis adducts. In this context, boron-doped nanographene **1** is expected to form 1 : 2 Lewis acid–base adducts (Scheme 2), and either *cis*- or *trans*-coordination modes are possible. Even more importantly, the formation of such Lewis adducts can be regarded as a model for chemical adsorption processes on boron-doped graphene. The potential of boron-doped graphene for chemisorption has recently generated increasing attention, as this aspect may be beneficial for various applications, including chemical sensing, hydrogen storage, and surface-enhanced Raman scattering.^19^ Although several theoretical studies have addressed this issue, experimental insight still remains to be gathered.6*a* Therefore, we now evaluated the Lewis acidity of **1** in order to gain a quantitative understanding of the chemisorption properties of the local BC_3_ sites in the boron-doped nanographene.

**Fig. 2:**
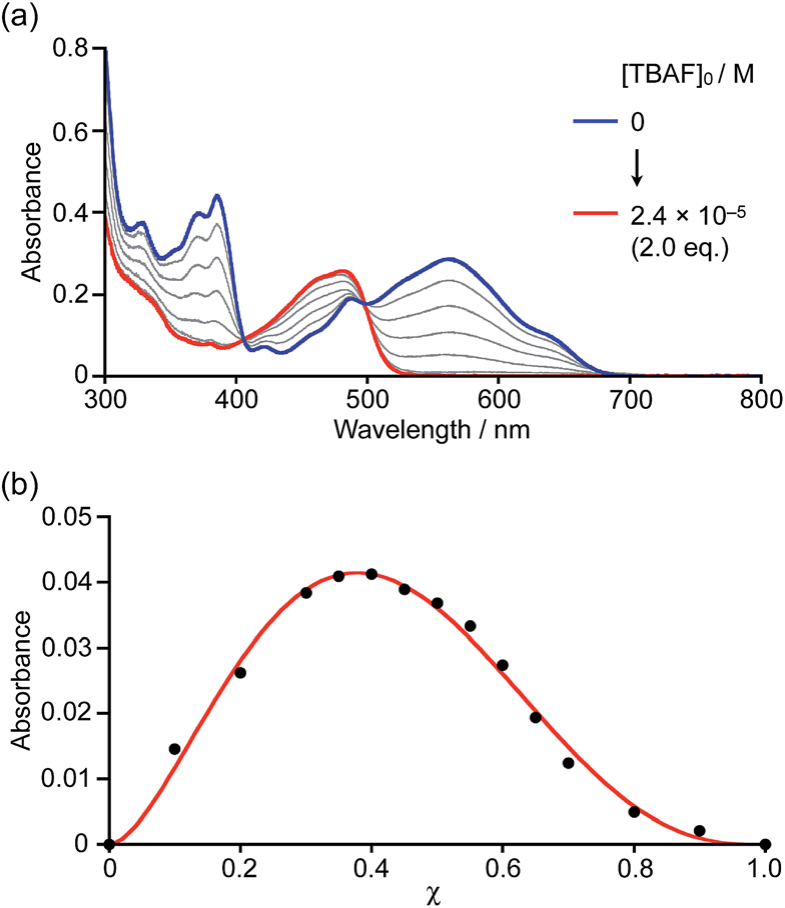
(a) Spectral absorption change upon addition of TBAF to a THF solution of **1a** (1.2 × 10^–5^ M) and (b) Job plot of **1a** with TBAF, where the parameter *χ* is defined by *χ* = [**1a**]_0_/([**1a**]_0_ + [TBAF]_0_). In this experiment: [**1a**]_0_ + [TBAF]_0_ = 1.2 × 10^–5^ M.

Initially, boron-doped nanographene **1a** was titrated with fluoride ions, *i.e.* with a strong Lewis base. Upon addition of tetra-*n*-butylammonium fluoride (TBAF) to a THF solution of **1a**, the color of the solution changed from purple to yellow. The corresponding UV-visible absorption spectra showed that the absorption bands of **1a** around 390 nm and 580 nm disappeared, and that a new band appeared around 470 nm with increasing concentrations of fluoride ions (Fig. 2a). The spectral change continued until the addition of two equivalents of TBAF. The formation of 1 : 2 complexes was confirmed by a Job plot analysis. When [**1a**]_0_ + [TBAF]_0_ is kept constant, the absorbance of the Lewis adducts at 474 nm produces a maximum at a ratio of *χ* = 0.37, indicative of a 1 : 2 coordination mode (Fig. 2b). Even with weaker Lewis bases, such as pyridine, 4-methylpyridine (MePy), or 4-dimethylaminopyridine (DMAP), similar spectral changes were observed after the addition of an excess of these bases (Fig. S5–S7†). The ^11^B NMR spectrum of **1b** in pyridine-*d*_5_ showed a broad signal at –2.78 ppm, supporting the generation of a tetracoordinate boron compound.^20^

Based on the titration of **1a** with pyridine monitored by the UV-visible absorption spectroscopy and the spectral analysis using the nonlinear least-square fitting procedure,^21^ binding constants of *K*_1_ = 23 M^–1^ and *K*_2_ = 16 M^–1^ were determined for the first and second coordination steps at the two BC_3_ sites (Fig. S10†). Moreover, binding constants with MePy and DMAP were also determined to be *K*_1_ = 150 M^–1^, *K*_2_ = 72 M^–1^ and *K*_1_ = 3.8 × 10^4^ M^–1^, *K*_2_ = 2.3 × 10^4^ M^–1^, respectively (Fig. S11 and S12†). These results demonstrate that there is no significant allosteric effect for these systems. Notably, although the absorption spectra seemingly changed with isosbestic points during the titration, the careful inspection of the enlarged spectra showed the absence of clear isosbestic point (Fig. S3 and S8†), indicative of sequent formation of the mono and bis Lewis adducts. In the titration of **1a** with DMAP, the nonlinear least-square fitting of the absorption spectra allowed us to estimate the absorption spectrum of the mono Lewis adduct **1a·DMAP** (Fig. S13†), in which the onset of the absorption band reaches longer wavelength compared to those of **1a** and the bis Lewis adduct **1a·(DMAP)_2_**. DFT structural optimization and TD-DFT calculations supported the smaller energy S_0_ → S_1_ transition of the mono Lewis adduct due to its donor–acceptor type electronic structure (Fig. S24†).

**Fig. 3:**
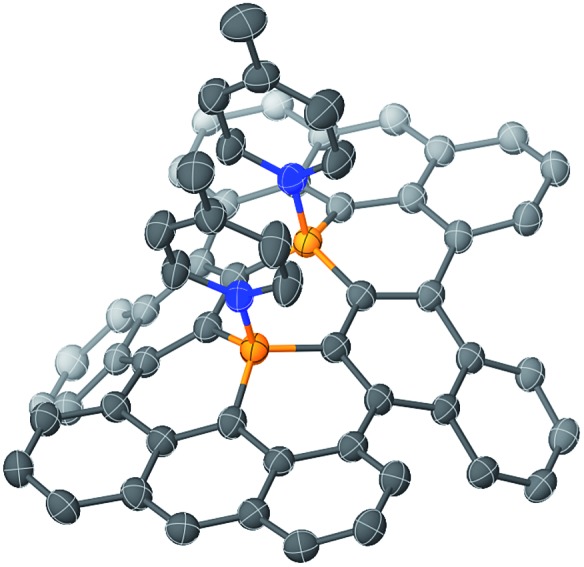
Molecular structure of **1b·(MePy)_2_** (thermal ellipsoids set at 50% probability; butoxy substituents and hydrogen atoms are omitted for clarity). Orange: boron; blue: nitrogen.

The structure of B–N Lewis adduct **1b·(MePy)_2_** was determined by single crystal X-ray diffraction analysis (Fig. 3). Single crystals of **1b·(MePy)_2_** were obtained from the slow diffusion of heptane into a solution of **1b** in MePy. Notably, two molecules of MePy coordinate to the boron atoms in a *cis* fashion, *i.e.* on the same side of the honeycomb sheet. The two pyridine rings are closely aligned exhibiting an N···N distance of 3.23 Å, while the diborapentacene moiety was highly contorted. A dihedral angle of 89° between the terminal benzene rings in the diborapentacene substructure and the B–N bond lengths of 1.665(2) Å and 1.664(2) Å were observed. Upon coordination, slightly elongated B–C bonds (0.063–0.090 Å) relative to those in **1b** were observed. The angle sums around both boron centers are 333°, which corresponds to a tetrahedral character of 86%.^22^ DFT calculations (B3LYP/6-311+G**) suggested that the *cis*-complex should be formed selectively in solution, as the *cis*-isomer is by 6.1 kcal mol^–1^ energetically more favorable than the *trans*-isomer (Fig. S23†).

**Fig. 4:**
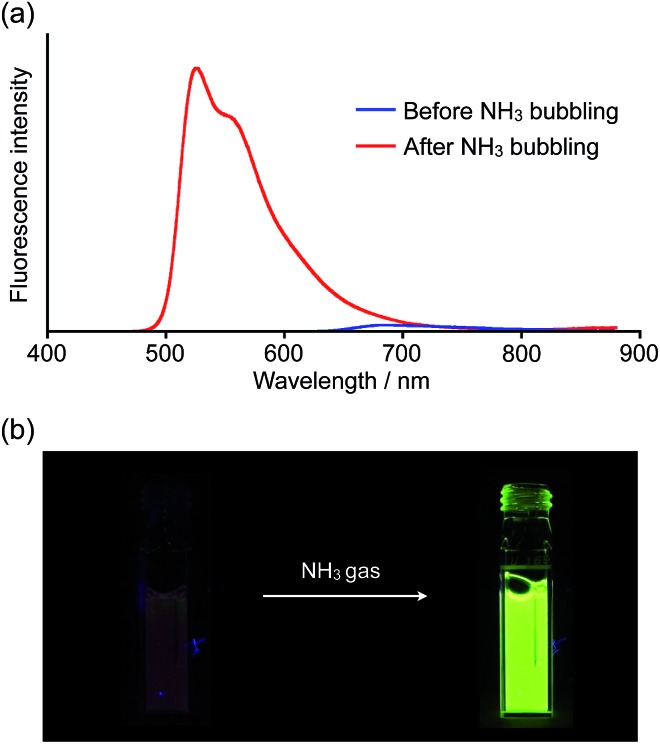
(a) Spectral change of the fluorescence upon bubbling NH_3_ through a THF solution of **1a** (*λ*_ex_ = 440 nm) and (b) corresponding turn-on fluorescence response.

Inspired by recent theoretical studies that proposed boron-doped graphene as a promising material for gas sensors,19a,e,f we exposed a solution of **1a** to gaseous NH_3_. When NH_3_ was bubbled for a few seconds through a THF solution of **1a**, the solution color changed from purple to yellow and a corresponding change of the absorption spectrum was observed (Fig. S9†). We also witnessed a drastic change of the fluorescence properties as a result of this process (Fig. 4 and movie in the ESI†). In the absence of gaseous NH_3_, the THF solution of **1a** showed only weak fluorescence in the visible-NIR region (*Φ*_F_ = 0.03), but after exposure to NH_3_, bright yellowish green fluorescence was observed at 526 nm (*Φ*_F_ = 0.42).

Remarkable turn-on fluorescence was also observed in the titration experiments with strong bases, such as TBAF and DMAP. The significant fluorescence response was completed with the low concentration of [TBAF] = 2.4 × 10^–5^ M or [DMAP] = 2.3 × 10^–3^ M when using a THF solution of **1a** (1.2 × 10^–5^ M) (Fig. S4 and S7†). On the other hand, only weak fluorescence emerged even upon the addition of the high concentration of [pyridine] = 4.1 × 10^–1^ M or [MePy] = 3.4 × 10^–1^ (Fig. S5 and S6†). Since the onset of the absorption band for the mono Lewis adduct reaches 700 nm (Fig. S13†), the increased yellowish green fluorescence around 530 nm should be attributed to the emission from the bis Lewis adduct, rather than the mono Lewis adduct, which may be non-emissive. Consequently, only the strong Lewis bases with high binding constants can give rise to the conspicuous fluorescence response. This finding would be important for the development of nanographene-based sensors.

### Redox properties

The electrochemical properties of boron-doped nanographene **1a** were previously examined by cyclic voltammetry in THF. Regardless of the scan rates, two reversible redox waves were observed for reduction processes at *E*_1/2_ = –1.45 V and –1.66 V (*vs.* Fc/Fc^+^), in addition to redox waves for an oxidation process at *E*_1/2_ = +0.62 V. The multi-step reduction process was not observed in a previously synthesized singly boron-embedded polycyclic aromatic system within the measured potential window.14*c* Thus, the second boron atom in **1a** is crucial for the storage of two electrons on the polycyclic aromatic framework.^23^

**Fig. 5:**
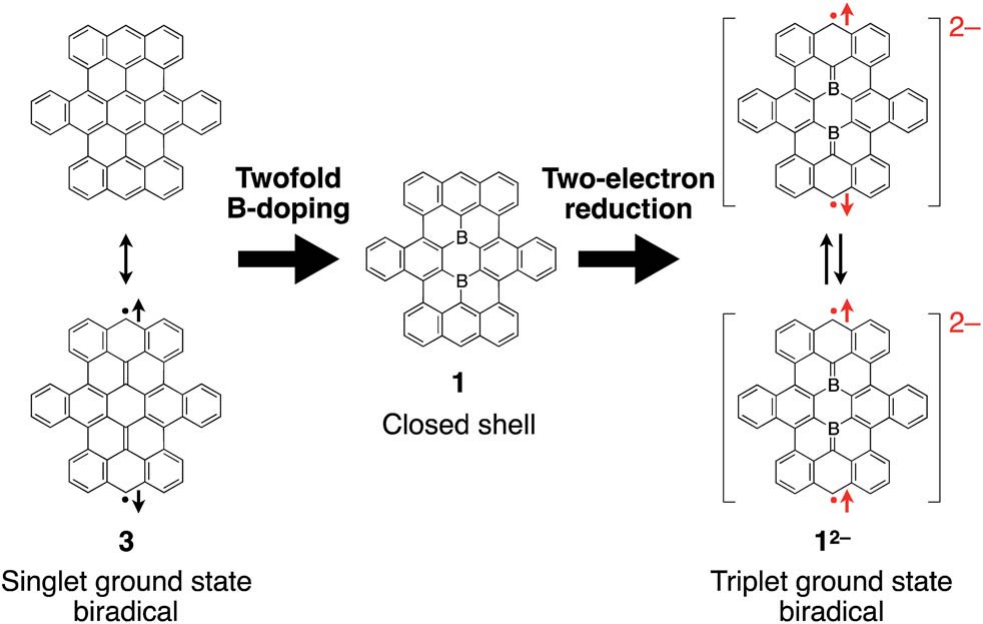
Isoelectronic relationship between parent nanographene **3** and reduced boron-doped nanographene **1^2–^**.

The two boron atoms also play an important role for the formation of a stable closed-shell structure of the boron-doped nanographene skeleton in **1**. Based on previous theoretical calculations, parent nanographene **3** with zigzag edges was expected to exhibit a singlet biradical character,14*d* similar to teranthene, which was recently reported by Kubo and co-workers.^24^ Notably, the two-electron reduced species **1^2–^** is isoelectronic with parent nanographene **3** (Fig. 5). The biradical character of the dianionic species **1^2–^** is therefore an interesting subject to study, which should depend on the coupling of the unpaired electrons across the boron-doped polycyclic framework.

**Fig. 6:**
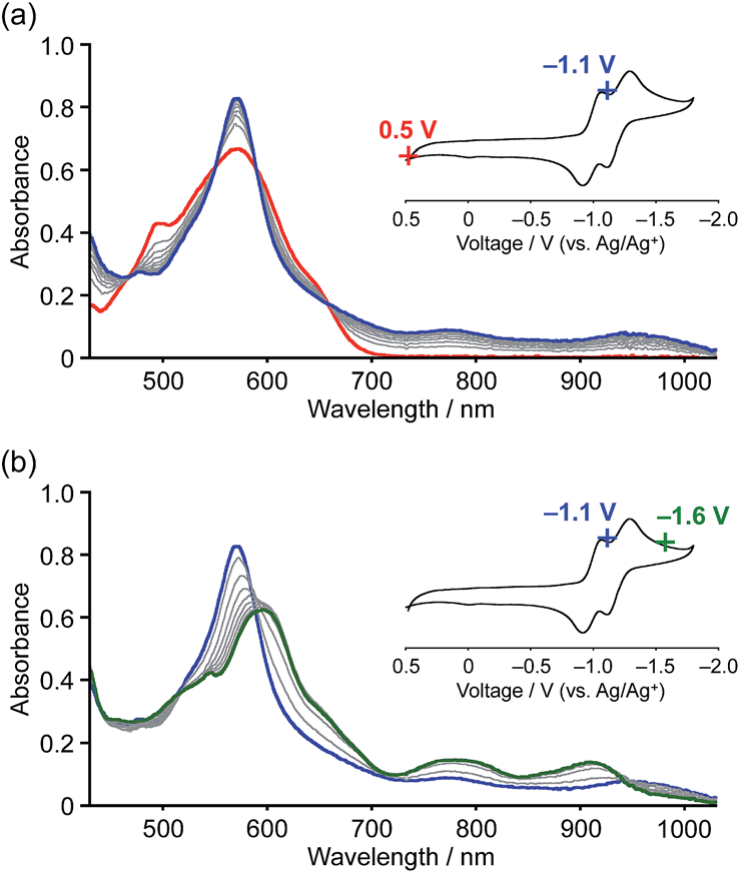
*In situ* visible-NIR absorption spectra of **1a** during electrochemical reduction in *o*-dichlorobenzene (3.4 × 10^–4^ M). Working electrode: Pt mesh; reference electrode: Ag/AgNO_3_; electrolyte: 0.1 M [(*n*Bu)_4_N][PF_6_].

The reduction processes were monitored *in situ* by visible-NIR absorption spectroscopy (Fig. 6). The first one-electron reduction occurred at *E*_app_ = –1.1 V (*vs.* Ag/Ag^+^) and the absorption spectrum gradually changed accordingly, *i.e.* new absorption bands appeared at 790 and 950 nm with clear isosbestic points. The observed low-energy absorption bands reaching the wavelength of 1000 nm are consistent with an open-shell character of the radical anion.^25^ The second reduction occurred upon applying a voltage of *E*_app_ = –1.6 V, resulting in the appearance of broad absorption bands at 600, 790, and 920 nm. The distinct two-step spectral change is indicative of the generation of dianionic species **1a^2–^**. Reverting the applied voltage to *E*_app_ = +0.5 V resulted in a recovery of the absorption spectrum of the neutral state (Fig. S14†).

We were also able to prepare dianion **1a^2–^** by chemical reduction, even though initial attempts to reduce **1b** by treatment with an excess of K metal in THF failed due to unexpected over-reduction. X-ray crystallographic analysis revealed the generation of the corresponding trianionic monoradical of the parent polycyclic framework, in which all the peripheral alkoxy groups were removed *via* cleavage of the C(sp^2^)–O bonds (Fig. S21†).^26^ Subsequently, we decided to use the milder reducing agent [CoCp*_2_] (Cp* = 1,2,3,4,5-pentamethylcyclopentadienyl) for the preparation of the dianion. Upon addition of 2.5 equivalents to a solution of **1a** in *o*-dichlorobenzene, the color of the solution changed from purple to green within 5 min. The visible-NIR absorption spectrum of the solution, displaying broad absorption bands at 605, 790, and 900 nm, is consistent with that of the electrochemically generated dianion **1a^2–^** (Fig. S15 and S16†). Therefore, we concluded that the dianion 2[CoCp*_2_]^+^·1a^2–^ was successfully generated.

**Fig. 7:**
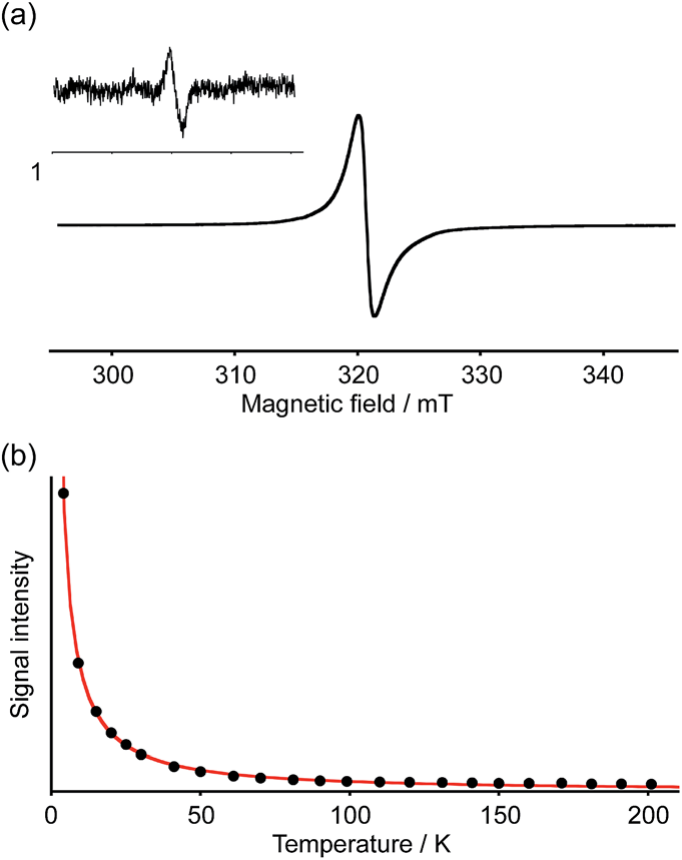
(a) ESR spectrum of 2[CoCp*_2_]^+^·1a^2–^ in a frozen *o*-dichlorobenzene matrix at 4 K. Inset: the weak forbidden Δ*m*_S_ = ±2 resonance at 4 K; (b) temperature dependence of the ESR signal intensity of 2[CoCp*_2_]^+^·1a^2–^ and curve-fitting with the Bleaney–Bowers equation.

The spin multiplicity of the dianion was investigated by variable-temperature ESR spectroscopy. ESR samples were prepared by treatment of **1a** with 3.0 equivalents of [CoCp*_2_] in *o*-dichlorobenzene. Both [Co(iii)(Cp*)_2_]^+^ and unreacted [Co(ii)Cp*_2_] are ESR-silent and should accordingly not interfere with the ESR studies.^27^ As the dianion 2[CoCp*_2_]^+^·1a^2–^ was found to be unstable in solution at room temperature, ESR measurements were carried out in frozen solution. The ESR spectra at 4 K exhibited a broad featureless signal in the Δ*m*_S_ = ±1 region centered at *g* = 2.0019, indicating an open-shell biradical character for 2[CoCp*_2_]^+^·1a^2–^ (Fig. 7a). Spin–spin interaction was confirmed by the observation of a weak forbidden signal in the half-field region (Δ*m*_S_ = ±2). In order to determine the spin multiplicity of 2[CoCp*_2_]^+^·1a^2–^ in the ground state, the temperature dependence of the resonance intensity for Δ*m*_S_ = ±1 was examined in the 4–200 K range (Fig. 7b). The observed decreasing resonance intensity as a function of increasing temperature demonstrated that the dianion possesses a triplet ground state. Based on a curve fitting using the Bleaney–Bowers equation, an energy gap between the singlet and triplet states (Δ*E*_ST_) of 0.45 kJ mol^–1^ was estimated.^28^ Accordingly, the triplet and singlet biradical states can be considered energetically close to each other. Upon warming the sample to room temperature, the ESR signal disappeared due to degradation of **1a^2–^**.

**Fig. 8:**
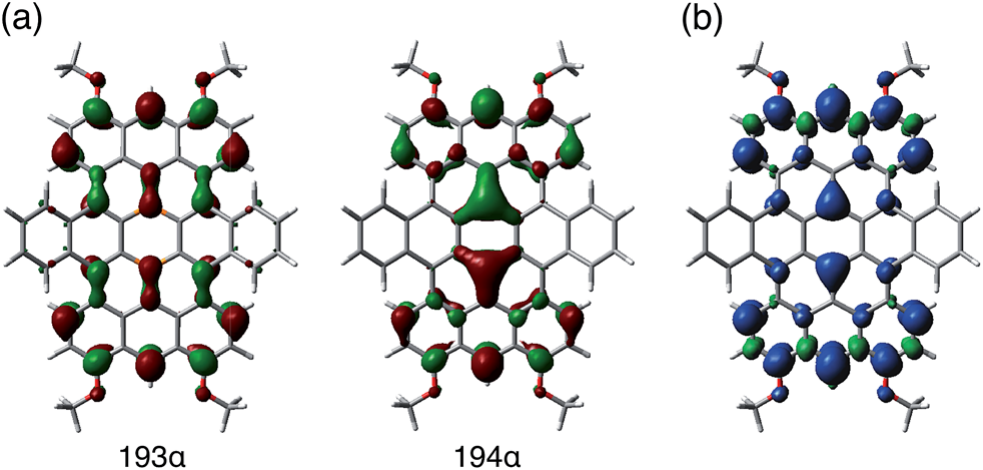
(a) SOMOs and (b) calculated (UB3LYP/6-311+G**) spin density distribution of the model compound **1b′^2–^**, in which the peripheral substituents of **1b** were replaced by methoxy groups for simplicity.

The results of a theoretical study on the biradical character of model compound **1b′**, in which the peripheral butoxy substituents of **1b** were replaced with methoxy groups for simplicity, were in good agreement with the experimental results. DFT calculations (UB3LYP/6-311+G**) using the broken-symmetry method demonstrated that the energy level of the triplet state was slightly lower than that of the singlet biradical state, and the energy gap Δ*E*_ST_ was calculated to be 0.38 kJ mol^–1^ (Fig. S25†).^29^ As a result, despite being isoelectronic to neutral carbon analogue **3**, dianion **1^2–^** has a different spin multiplicity in the ground state. In order to elucidate the origin of this difference, we reconsidered the molecular orbitals of neutral **1** (closed shell) and compared them with those of dicationic **3^2+^** (closed shell).14*d* Whereas the LUMO and LUMO+1 of **3^2+^** have almost identical spatial distributions but are different in energy (Δ*E* = 0.31 eV), the corresponding orbitals of **1** show a remarkably different distribution with almost equivalent energy levels (Δ*E* = 0.01 eV). These results suggest that the replacement of two carbon atoms with boron gives rise to a significant perturbation of the LUMO and LUMO+1 in the closed shell molecule prior to insertion of two electrons. The twofold reduction of **1** to **1^2–^** corresponds to a placement of two spins in the energetically similar LUMO and LUMO+1 according to Hund's rule. As a result, and in contrast to the singlet biradical ground state of **3**, **1^2–^** adopts a triplet biradical ground state. The spatial distribution of the LUMO and LUMO+1 of **1** corresponds to those of the SOMOs in the triplet biradical state of **1^2–^**. One of the SOMOs is delocalized over the zig-zag edges, while the other is located over the central boron atoms and the zig-zag edges. The spin distribution map of the triplet state is consistent with a delocalization of the SOMOs (Fig. 8).

### Battery electrode performance

Graphene has attracted considerable attention as a promising electrode material, mostly due to its outstanding electric and thermal conductivity and remarkable mechanical flexibility. Recently, Cheng and co-workers investigated boron-doped graphene as an anode material in Li-ion batteries and demonstrated its superior performance relative to pristine graphene.^3^ This report, regarding the positive effect of boron doping on battery performance, encouraged us to evaluate the properties of boron-doped nanographene **1** as an electrode material. A desirable advantage of relatively small molecules as electrode materials is the possibility to implement custom-tailored structural modifications at the molecular level.^16^,^30^

The electrode performance of boron-doped nanographene **1** was evaluated using a half-cell with a Li metal counter electrode. The working electrode consisted of 10 wt% boron-doped nanographene **1a** or **1b** (active material), 70 wt% carbon black (conductive agent), and 20 wt% polyvinylidene difluoride (PVDF, binder). A 1.0 M solution of Li[PF_6_] in diethyl carbonate/ethylene carbonate was used as an electrolyte solution.

Charge/discharge tests were carried out at a constant current (1 mA) in the 1.5–4.0 V (*vs.* Li/Li^+^) voltage range. In light of the cyclic voltammogram of **1c** (Fig. S17†), boron-doped nanographenes **1a** and **1b** were expected to exhibit a reversible two-electron reduction process in this voltage range. The voltage profile of both batteries showed a gentle curve, and distinct plateau regions were not observed. For **1a** and **1b**, first discharge capacity values of 111 mA h g^–1^ and 160 mA h g^–1^ were observed, respectively (Fig. S18†). As the peripheral alkoxy substituents are not expected to participate in the Li-ion storage because of the inert redox properties in this voltage range,^31^ compound **1b**, bearing more compact substituents relative to **1a**, displayed a higher specific capacity.

**Fig. 9:**
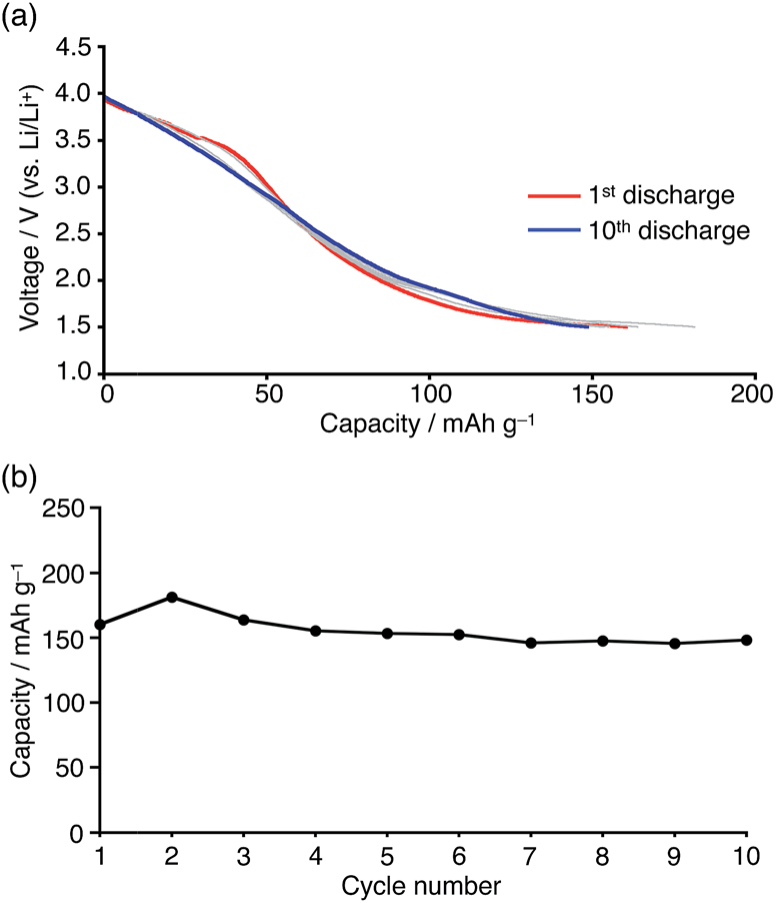
(a) Discharge profile and (b) cycle performance of Li batteries containing boron-doped nanographene **1b** at a constant current density of 0.5 mA cm^–2^ in the 1.5–4.0 V voltage range (*vs.* Li/Li^+^).

In order to evaluate the cycle stability, the battery containing **1b** was subjected to repeated charge/discharge cycles at a constant current density of 0.5 mA cm^–2^ in the 1.5–4.0 V (*vs.* Li/Li^+^) voltage range. The obtained voltage profile did not show significant changes and a discharge capacity of 150 mA h g^–1^ was retained even after 10 cycles, although the average discharging voltage is low when **1b** would be used for cathode materials (Fig. 9a). In light of the degradation of the reduced boron-doped nanographene in solution, confirmed by the electrochemical study, the stable cycle performance should be attributed to its low solubility, which should suppress dissolution of the material into the electrolyte solution.^30^ Based on the theoretical capacity (29 mA h g^–1^ per electron) and considering the molecular weight of **1b**, *ca.* 5–6 electrons/molecule were estimated to be involved in the charge/discharge process. Since a reference cell without active material showed only small capacity (13 mA h g^–1^) in the same voltage range, electron storage by the carbon black and PVDF is limited (Fig. S19†). Thus, the total capacity of the boron-doped nanographene material is larger than the two-electron storage observed in the cyclic voltammetry, which should most likely be attributed to the super capacitor effect frequently observed in graphene materials.^32^

## Conclusions

We have presented a comprehensive study on fundamental and characteristic properties and functions of boron-doped nanographenes containing two boron atoms. We have discussed their Lewis acidity, redox properties, and battery electrode performance. The boron atoms in the honeycomb framework act as Lewis acidic coordination sites and they form Lewis adducts with various Lewis bases. The change in coordination number of the boron atoms results in significant perturbations, not only with respect to the molecular structure, but also with regard to the electronic structure, which induced turn-on fluorescence response to gaseous NH_3_. The two-electron reduction of such twofold boron-doped nanographenes furnished a dianionic species, which exhibited a biradical character with a triplet ground state. This is in stark contrast to the singlet biradical character expected for the corresponding pristine carbon-based nanographene. Boron doping followed by the electron reduction may thus be considered an effective way to modulate the spin multiplicity of nanographenes. We have also examined the pivotal role of the peripheral substituents on the nanographene skeleton. Replacing the bulky mesityloxy groups with sterically less demanding alkoxy groups resulted in the formation of a more densely packed structure. When the boron-doped nanographenes were utilized as an active material for Li battery electrodes, the increased specific capacity was attained with the smaller substituents. A stable charge/discharge performance in the 1.5–4.0 V voltage range was displayed without any significant degradation even after 10 cycles. In conclusion, the precisely defined atomic structure of **1** enabled us to accurately elucidate the impact of boron doping on the characteristic properties and the functionality of nanographene materials at the molecular level. These findings should hence provide important fundamental guidelines for the design of advanced nanographene materials.

## Supplementary Material

Supplementary information

Supplementary movie

Crystal structure data
