# Supplementary material for: Boron-doped nanographene: Lewis acidity, redox properties, and battery electrode performance
Source: Chem Sci. 2015 Sep 24;7(1):219–27. doi: 10.1039/c5sc02246k (PMC5952319; doi:10.1039/c5sc02246k)
Supplement: Supplementary file 1 [file SC-007-C5SC02246K-s001.pdf]

Electronic Supplementary Material (ESI) for Chemical Science.
This journal is © The Royal Society of Chemistry 2015

Electronic Supplementary Information

Boron-Doped Nanographenes: Lewis Acidity, Redox Properties, and Battery Electrode Performance

Shinichiro Osumi, Shohei Saito,* Chuandong Dou, Kyohei Matsuo, Keita Kume, Hirofumi Yoshikawa, Kunio Awaga and Shigehiro Yamaguchi*

Department of Chemistry, Graduate School of Science and Institute of Transformative Bio-Molecules (WPI-ITbM), Nagoya University, Furo, Chikusa, Nagoya 464-8602, Japan

Japan Science and Technology Agency (JST), CREST, Furo, Chikusa, Nagoya 464-8602, Japan

Contents

1. Experimental details S1
2. Photophysical properties of **1c** S5
3. Chemical stability S5
4. Spectral titrations of **1a** with various Lewis bases S6
5. Electrochemical reduction of **1a** S12
6. Chemical reduction S13
7. Variable temperature ESR analysis of $\mathbf{2[CoCp^*_2]^+\cdot 1a^{2-}}$ S15
8. Cyclic voltammetry of **1c** S16
9. Evaluation of battery electrode performance S17
10. X-ray crystallographic analyses S18
11. Theoretical calculations S21
12. References S25
13. NMR spectra S26

1. Experimental details

General. Melting points (mp) were determined with a Yanaco MP-S3 instrument. ^{1}H, ^{13}C, and ^{11}B NMR spectra were recorded with a JEOL AL-400 (400 MHz for ^{1}H, 100 MHz for ^{13}C, and 128 MHz for ^{11}B) spectrometer in $CDCl_3$, CD_2Cl_2, 1,1,2,2-tetrachloroethane-d_2, chlorobenzene-d_5, or pyridine-d_5. Chemical shifts are reported in δ ppm using $CHCl_3$ (7.26 ppm), CH_2Cl_2 (5.32 ppm), chlorobenzene (6.96, 6.99, and 7.14 ppm), or pyridine (7.22, 7.58, and 8.74 ppm) for ^{1}H NMR spectra and $CDCl_3$ (77.16 ppm), CD_2Cl_2 (53.84 ppm), or 1,1,2,2-tetrachloroethane-d_2 (73.78 ppm) for ^{13}C NMR spectra as an internal standard. The external standard of $BF_3 \cdot OEt_2$ was used for ^{11}B NMR spectra. Mass spectra were measured with a Bruker Daltonics micrOTOF Focus spectrometer with the ionization method of APCI, ESI, or a JEOL JMS-700 (FAB-MS). UV-visible absorption spectra were recorded on a Shimadzu UV-3510 spectrometer. Fluorescence spectra were recorded on a Hitachi F-4500 spectrometer. The absolute fluorescence quantum yields were determined with a Hamamatsu C9920-02 calibrated integrating sphere. Thin layer chromatography (TLC) was performed on plates coated with 0.25 mm thickness of silica gel $60F_{254}$ (Merck). Column chromatography was performed using silica gel PSQ 100B (Fuji Silysia Chemical). Commercially available solvents and reagents were used without further purification unless otherwise mentioned. Anhydrous THF, CH_2Cl_2, toluene and Et_2O were purchased from Kanto Chemicals and further purified using Glass Contour Solvent System (Nikko Hansen & Co., Ltd.). 4-Methylpyridine was distilled prior to use. *o*-Dichlorobenzene was distilled over CaH_2 and degassed by freeze-pump-thaw cycles. NH_3 gas was prepared by heating a 28% NH_3 aqueous solution. Compounds **4**[S1] and **6**[S2] were prepared according to the literature procedures. All reactions were performed under a nitrogen atmosphere.

Scheme S1.

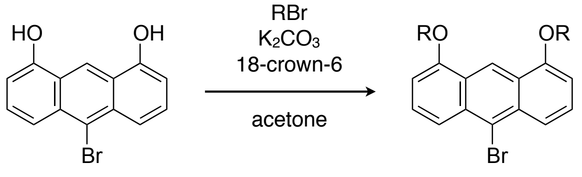

10-Bromo-1,8-dibutoxyanthracene (5b). This compound was prepared according to the published procedure.[S1] To a solution of **4** (1.60 g, 5.53 mmol), potassium carbonate (2.29 g, 16.6 mmol), and 18-crown-6 (0.31 g, 1.17 mmol) in acetone (500 mL) was added 1-bromobutane (1.78 mL, 16.6 mmol). The reaction mixture was heated at 70 °C for 4 days. The resulting mixture was cooled to ambient temperature and then filtered. The filtrate was concentrated under reduced pressure. The mixture was purified by silica gel column chromatography using hexane/CH_2Cl_2 (10/1, R_f = 0.28) as an eluent to afford **5b** as a yellow solid (1.53 g, 2.45 mmol, 47%). mp: 130.6–131.2 °C. ^{1}H NMR (400 MHz, $CDCl_3$): δ 9.41 (s, 1H), 8.03 (d, J = 8.8 Hz, 2H), 7.48 (dd, J_1 = 7.6 Hz, J_2 = 8.8 Hz, 2H), 6.76 (d, J = 7.6 Hz, 2H), 4.22 (t, J = 6.0 Hz, 4H), 2.02–1.95 (m, 4H), 1.75–1.66 (m, 4H), 1.08 (t, J = 7.6 Hz, 6H). ^{13}C NMR (100 MHz, $CDCl_3$): δ 155.5, 132.0, 127.7, 125.2, 121.3, 119.6, 116.7, 102.6, 68.3, 31.6, 19.8, 14.2. HRMS (APCI): Calcd for $C_{22}H_{25}BrO_2$: 400.1032 (M^+). Obsd. 400.1028.

10-Bromo-1,8-di(dodecyloxy)anthracene (5c). This compound was prepared according to the published procedure.[S1] To a solution of **4** (1.50 g, 5.19 mmol), potassium carbonate (2.15 g, 15.6 mmol), and 18-crown-6 (0.27 g, 1.04 mmol) in acetone (450 mL) was added 1-bromododecane (3.74 mL, 15.6 mmol). The reaction mixture was heated at 65 °C for 4 days. The resulting mixture was allowed to cool to ambient temperature and then filtered. The filtrate was concentrated under reduced pressure. The mixture was purified by silica gel column chromatography using hexane/AcOEt (40/1, R_f = 0.52) as an eluent to afford **5c** as a yellow solid (1.53 g, 2.45 mmol, 47%). mp: 73.5–74.2 °C. ^{1}H NMR (400 MHz, $CDCl_3$): δ 9.40 (s, 1H), 8.03 (d, J = 8.8 Hz, 2H), 7.48 (dd, J_1 = 8.0 Hz, J_2 = 8.8 Hz, 2H), 6.75 (d, J = 7.2 Hz, 2H), 4.21 (t, J = 6.4 Hz, 4H), 2.04–1.97 (m, 4H), 1.68–1.61 (m, 4H), 1.45–1.26 (m, 32H), 0.88 (t, J = 6.4 Hz, 6H). ^{13}C NMR (100 MHz, $CDCl_3$): δ 155.6, 132.0, 127.6, 125.2, 121.4, 119.6, 116.8, 102.7, 68.6, 32.1, 29.9, 29.7, 29.5, 29.5, 26.5, 22.8, 14.2. HRMS (APCI): Calcd for $C_{38}H_{57}BrO_2$: 624.3536 (M^+). Obsd. 624.3533.

Scheme S2.

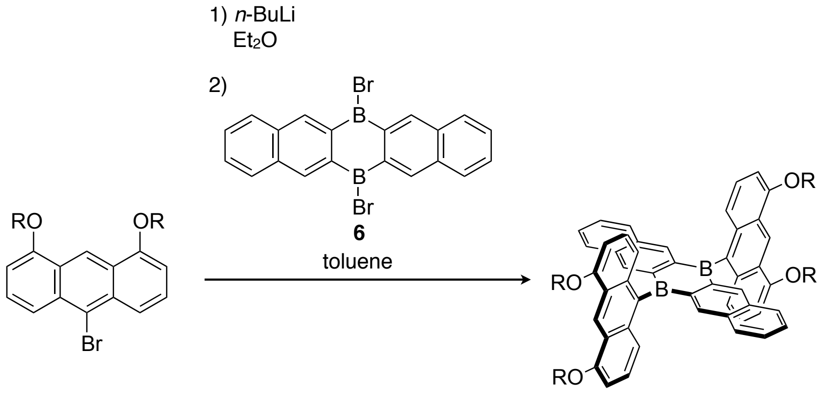

5b: R = C_4H_9
5c: R = $C_{12}H_{25}$

2b: R = C_4H_9
2c: R = $C_{12}H_{25}$

6,13-Bis(4,5-dibutoxy-9-anthryl)-6,13-dihydro-6,13-diborapentacene (2b). To a solution of **5b** (161 mg, 0.40 mmol) in Et_2O (23 mL) was added 1.6 M *n*-BuLi in hexane (0.30 mL, 0.40 mmol) dropwise at 0 °C. The reaction mixture was stirred at 25 °C for 25 min. After removing the solvent under reduced pressure, a suspension of **6** (86.5 mg, 0.20 mmol) in toluene (20 mL) was added slowly. The resulting mixture was stirred at 25 °C for 19 h, and the solvent was removed under reduced pressure. The crude product was washed with MeOH (60 mL) and hexane (50 mL) to afford **2b** as a light orange solid (149 mg, 0.162 mmol, 81%). mp: > 300 °C. ^{1}H NMR (400 MHz, $CDCl_3$): δ 9.59 (s, 2H), 8.01 (s, 4H), 7.48 (dd, J_1 = 6.4 Hz, J_2 = 3.2 Hz, 4H), 7.33–7.30 (m, 8H), 7.18 (dd, J_1 = 8.0 Hz, J_2 = 8.0 Hz, 4H), 6.75 (d, J = 7.2 Hz, 4H), 4.32 (t, J = 6.0 Hz, 8H), 2.12–2.06 (m, 8H), 1.87–1.77 (m, 8H), 1.17 (t, J = 7.2 Hz, 12H). ^{13}C NMR (100 MHz, 1,1,2,2-tetrachloroethane-d_2): δ 152.4, 139.9, 132.7, 132.1, 126.6, 125.0, 121.5, 121.1, 119.3, 112.3, 98.8, 64.6, 28.4, 16.6, 10.9. Two signals for the carbon atoms bonding to the boron atoms were not observed due to the quadrupolar relaxation of the boron atom. ^{11}B NMR spectrum was not obtained due to its poor solubility. MS (APCI): Calcd for $C_{64}H_{62}B_2O_4$: 916.4834 (M^+). Obsd. 916.4832.

6,13-Bis(4,5-didodecyloxy-9-anthryl)-6,13-dihydro-6,13-diborapentacene (2c). To a solution of **5c** (201 mg, 0.32 mmol) in ether (15 mL) was added 1.6 M *n*-BuLi in hexane (0.20 mL, 0.32 mmol) dropwise at 0 °C. The reaction mixture was stirred at 25 °C for 40 min. After removing the solvent under reduced pressure, the mixture was dissolved together with **6** (58.1 mg, 0.13 mmol) in toluene (10 mL). The resulting mixture was stirred at 25 °C for 19 h, and the solvent was removed under reduced pressure. The crude product was washed with MeOH (40

mL) and hexane (20 mL) to afford **2c** as a red solid (125 mg, 0.09 mmol, 70%). mp: 178.4–180.5 ℃. ^{1}H NMR (400 MHz, CD_2Cl_2): δ 9.55 (s, 2H), 8.00 (s, 4H), 7.52–7.50 (m, 4H), 7.37 (dd, J_1 = 6.4 Hz, J_2 = 3.2 Hz, 4H), 7.29 (d, J = 8.4 Hz, 4H), 7.23–7.17 (m, 4H), 6.77 (d, J = 7.2 Hz, 4H), 4.32 (t, J = 6.0 Hz, 8H), 2.13–2.06 (m, 8H), 1.79–1.72 (m, 8H), 1.57–1.30 (m, 64H), 0.89 (t, J = 6.8 Hz, 12H). ^{13}C NMR (100 MHz, CD_2Cl_2): δ 156.1, 143.3, 136.3, 135.7, 130.0, 129.0, 128.9, 125.2, 124.7, 122.6, 116.0, 102.6, 68.7, 32.4, 30.3, 30.2, 30.2, 30.0, 29.9, 29.9 26.9, 23.1, 14.3. Two signals for the carbon atoms bonding to the boron atoms were not observed due to the quadrupolar relaxation of the boron atom. ^{11}B NMR spectrum was not obtained due to its poor solubility. MS (ESI): Calcd for $C_{96}H_{126}B_2O_4$: 1366.9842 (M^+). Obsd. 1364.9839.

Tetrabutoxy-substituted boron-doped nanographene 1b. To a solution of **2b** (147 mg, 0.16 mmol) in CH_2Cl_2 (240 mL) cooled to 0 °C was added a solution of anhydrous $FeCl_3$ (400 mg, 2.46 mmol) in CH_3NO_2 (8 mL) dropwise. The mixture was stirred at 25 °C for 20 min. After addition of MeOH (200 mL), purple precipitates were collected by filtration. The crude product was purified by recrystallization from chlorobenzene to afford **1b** as a deep purple solid (27.8 mg, 0.03 mmol, 19%). mp: > 300 °C. ^{1}H NMR (400 MHz, chlorobenzene-d_5, 110 °C): δ 10.43 (s, 2H), 9.15 (d, J = 7.6 Hz, 4H), 9.12–9.10 (m, 4H), 7.71 (dd, J_1 = 7.6 Hz, J_2 = 3.2 Hz, 4H), 7.10–7.08 (m, 4H), 4.33 (t, J = 6.0 Hz, 8H), 2.05–2.00 (m, 8H), 1.76–1.71 (m, 8H), 1.12 (t, J = 7.2 Hz, 12H). ^{13}C and ^{11}B NMR spectra were not obtained due to its poor solubility. MS (FAB): Calcd for $C_{64}H_{54}B_2O_4$: 908.4208 (M^+). Obsd. 908.4223.

Tetradodecyloxy-substituted boron-doped nanographene 1c. To a solution of **2c** (28.0 mg, 0.021 mmol) in CH_2Cl_2 (30 mL) cooled to 0 °C was added a solution of anhydrous $FeCl_3$ (55.2 mg, 0.340 mmol) in CH_3NO_2 (1.2 mL) dropwise. After stirring at 0 °C for 4 h, a saturated aqueous $NaHCO_3$ solution was added. The mixture was extracted with CH_2Cl_2. The combined organic layer was washed with water and brine and dried over anhydrous Na_2SO_4. After concentration under reduced pressure, the mixture was purified by silica gel column chromatography using hexane/CH_2Cl_2 (1/1, R_f = 0.88) as an eluent to afford **1c** as a deep purple solid (9.3 mg, 7.0 μmol, 34%). mp: 232.0–233.0 °C. ^{1}H NMR (400 MHz, $CDCl_3$): δ 10.28 (s, 2H), 9.14 (d, J = 8.0 Hz, 4H), 9.03 (dd, J_1 = 5.6 Hz, J_2 = 3.6 Hz, 4H), 7.74 (dd, J_1 = 7.2 Hz, J_2 = 3.2 Hz, 4H), 7.26 (m, overlapped with residual $CHCl_3$), 4.49 (t, J = 6.0 Hz, 8H), 2.19–2.16 (m, 8H), 1.80–1.76 (m, 8H), 1.57–1.28 (m, 64H), 0.88 (t, J = 6.4 Hz, 12H). ^{13}C and ^{11}B NMR spectra were not obtained due to its poor solubility. MS (FAB): Calcd for $C_{96}H_{118}B_2O_4$:

1356.9216 (M^+). Obsd. 1356.9198.

B–N Lewis adduct of 1b with pyridine (1b·Py$_2$). ^{1}H NMR (400 MHz, pyridine-d_5): δ 9.72 (s, 2H), 9.19 (dd, J_1 = 6.4 Hz, J_2 = 3.2 Hz, 4H), 8.76 (overlapped with a signal of residual proton of pyridine-d_5), 7.66 (dd, J_1 = 6.4 Hz, J_2 = 3.2 Hz, 4H), 7.14 (d, J = 7.6 Hz, 4H), 4.34 (m, 4H), 4.23 (m, 4H), 1.90 (m, 8H), 1.67 (m, 8H), 1.02 (t, J = 7.6, 12H). ^{11}B NMR (128 MHz, pyridine-d_5): δ –2.78.

2. Photophysical properties of 1c

UV-visible absorption spectra were recorded on a Shimadzu UV-3510 spectrometer. Vis-NIR fluorescence spectra were recorded on a Fluorolog-3 spectrometer (HORIBA Jobin Yvon).

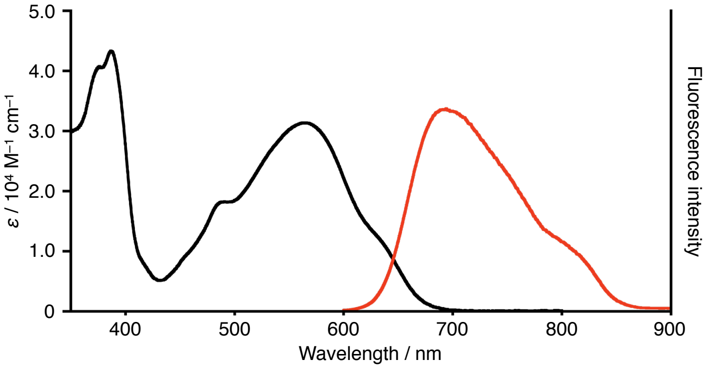

Figure S1. UV-visible absorption (black line) and vis-NIR fluorescence (red line, λ_{ex} = 570 nm) spectra of **1c** in CH_2Cl_2.

3. Chemical stability

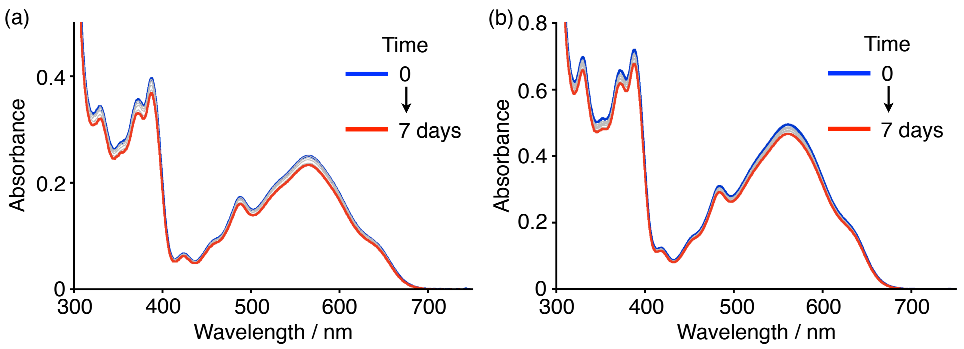

Figure S2. UV-visible absorption spectral changes of toluene solutions of (a) **1a** and (b) **1c** upon standing in air.

4. Spectral titrations of 1a with various Lewis bases

UV-visible absorption spectra were recorded on a Shimadzu UV-3510 spectrometer. Fluorescence spectra were recorded on a Hitachi F-4500 spectrometer. The absolute fluorescence quantum yields were determined with a Hamamatsu C9920-02 calibrated integrating sphere. NH_3 gas was prepared by heating a 28% NH_3 aqueous solution.

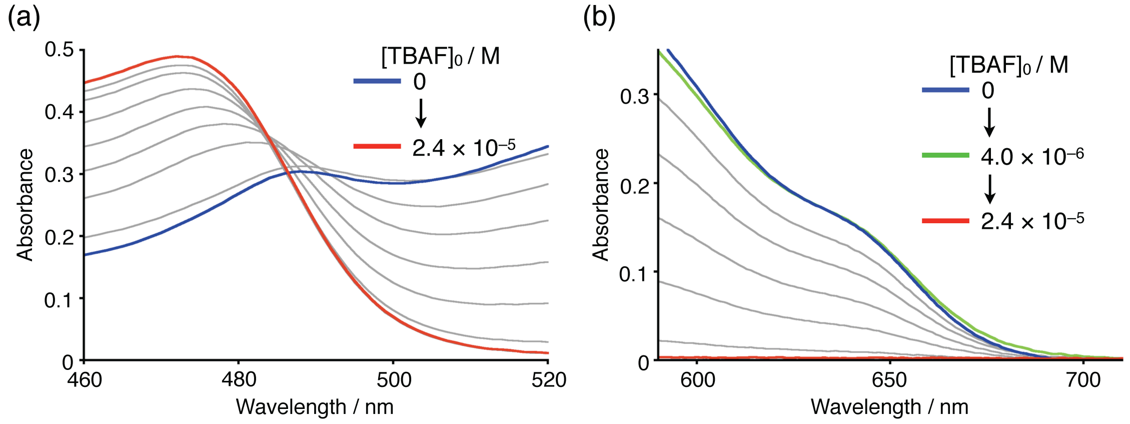

Figure S3. Enlarged UV-visible absorption spectra around (a) 490 nm and (b) 700 nm during the titration of **1a** with TBAF (see Figure 2a in the main text).

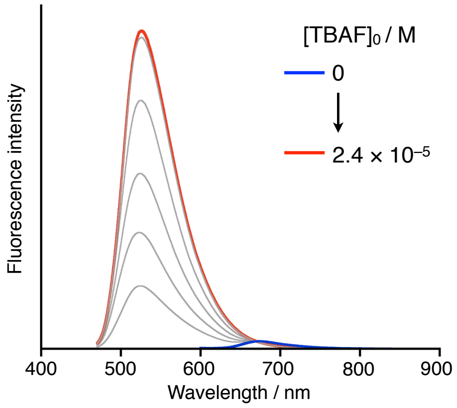

Figure S4. Fluorescence spectral change upon the addition of TBAF to a THF solution of **1a** (1.2×10^{-5} M, $\lambda_{ex} = 450$ nm).

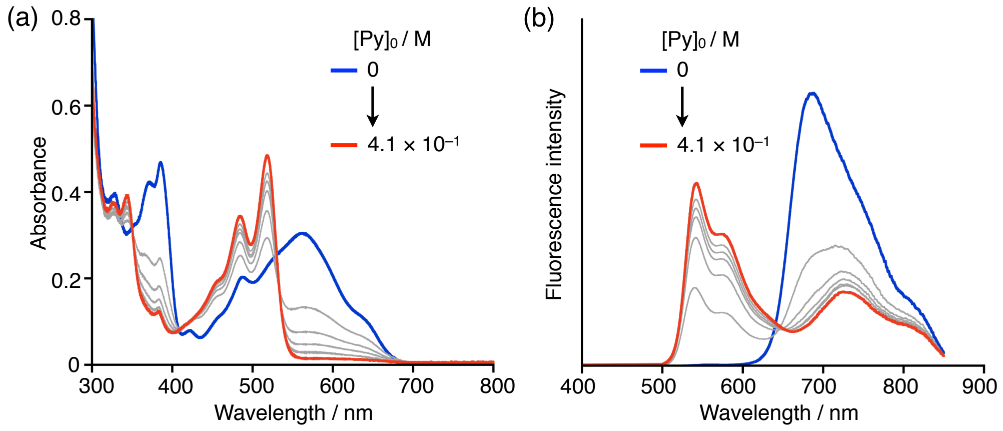

Figure S5. Spectral changes upon the addition of pyridine to a THF solution of **1a** (1.2×10^{-5} M): (a) UV-visible absorption spectra and (b) fluorescence spectra (λ_{ex} = 480 nm).

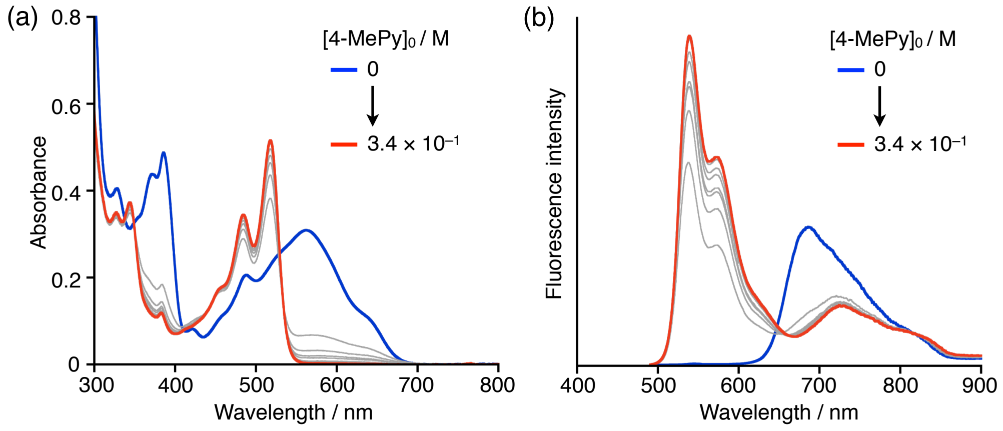

Figure S6. Spectral changes upon the addition of 4-methylpyridine to a THF solution of **1a** (1.2×10^{-5} M): (a) UV-visible absorption spectra and (b) fluorescence spectra (λ_{ex} = 470 nm).

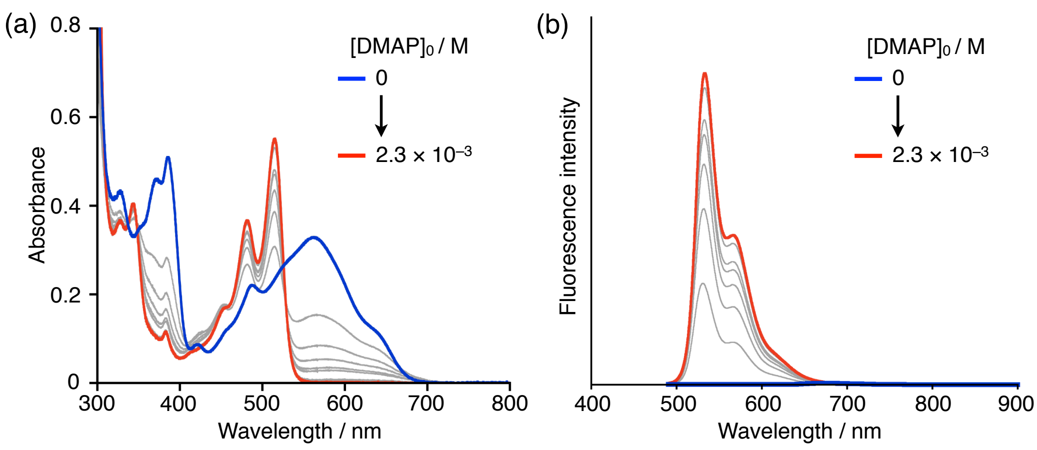

Figure S7. Spectral changes upon the addition of 4-dimethylaminopyridine (DMAP) to a THF solution of **1a** (1.2×10^{-5} M): (a) UV-visible absorption spectra; (b) fluorescence spectra (λ_{ex} = 470 nm).

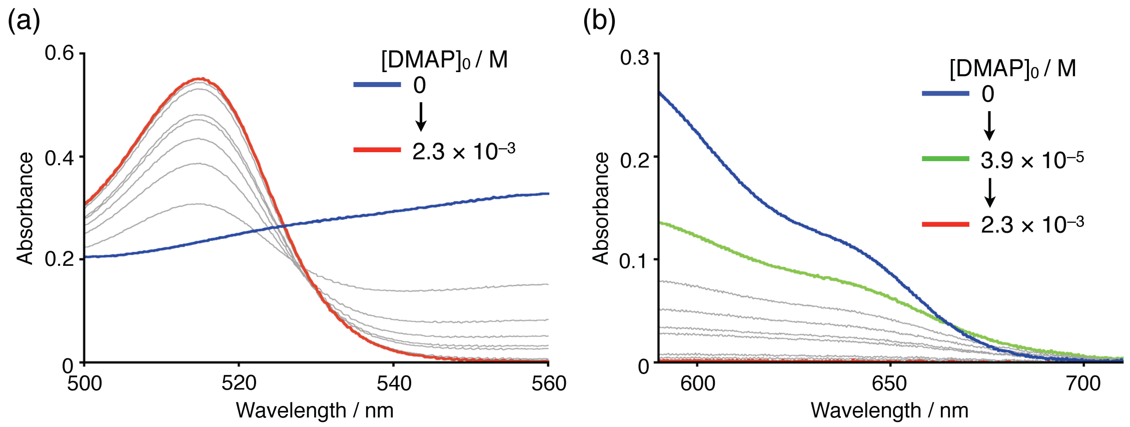

Figure S8. Enlarged UV-visible absorption spectra around (a) 520 nm and (b) 700 nm during the titration of **1a** with DMAP (see Figure S7a).

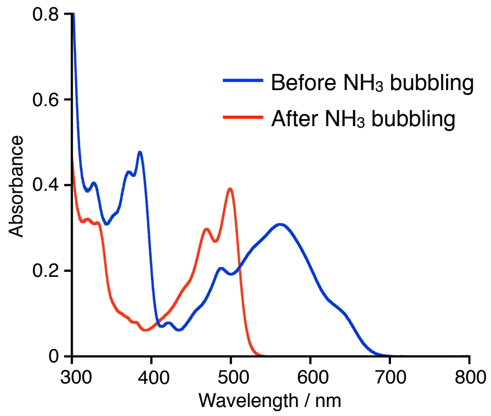

Figure S9. Absorption spectral change upon bubbling of NH_3 gas to a THF solution of **1a** (1.2×10^{-5} M).

Determination of the binding constants. The binding constants K_1 (= [**1a·LB**]/([**1a**][LB])) and K_2 (= [**1a·LB$_2$**]/([**1a·LB**][**LB**])) were determined by a nonlinear least-squares fitting of ΔA_{obs} (change of absorbance) × $[LB]_0$ (initial concentration of Lewis base) plot with the following equation.[S3]

$$\Delta A_{obs} = \frac{b[1a]\left(K_1 \Delta\varepsilon_1 [LB] + K_1 K_2 \Delta\varepsilon_2 [LB]^2\right)}{1 + K_1[LB] + K_1 K_2 [LB]^2}$$

where b is a constant while ε_1 and ε_2 are defined as ε(**1a·LB**)–ε(**1a**) and ε(**1a·LB$_2$**)–ε(**1a**), respectively. Nonlinear least-square fitting was performed using a spectral analysis program SPANA.[S4]

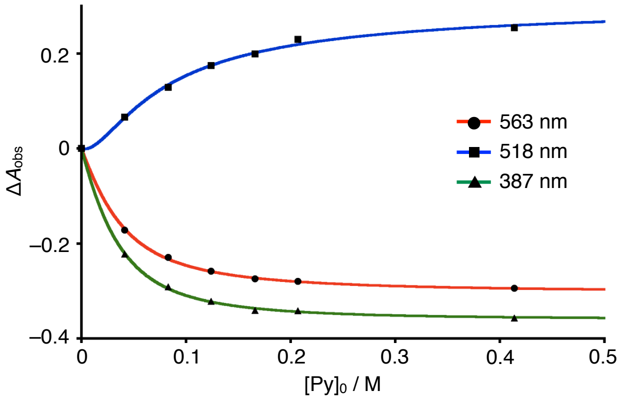

Figure S10. UV-visible absorption titration plot of **1a** with pyridine. The absorbances at 563, 518 and 387 nm of a toluene solution of **1a** (1.2×10^{-5} M) were monitored (black markers). The fitting curves were delineated for the determination of the binding constants of **1a** toward pyridine (lines). K_1 and K_2 were calculated to be 23 (± 2) M^{-1} and 16 (± 2) M^{-1}, respectively.

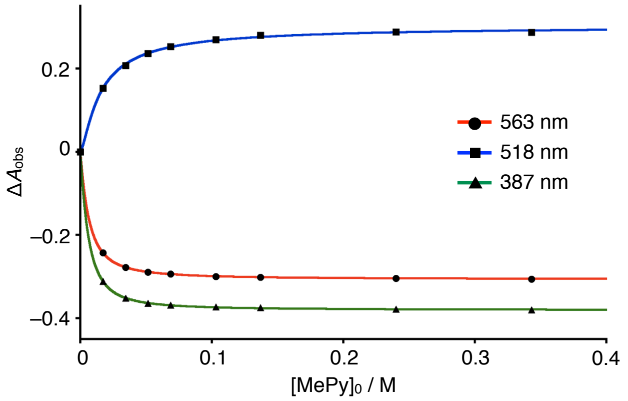

Figure S11. UV-visible absorption titration plot of **1a** with 4-methylpyridine. The absorbances at 563, 518 and 387 nm of a toluene solution of **1a** (1.2×10^{-5} M) were monitored (black markers). The fitting curves were delineated for the determination of the binding constants of **1a** toward 4-methylpyridine (lines). K_1 and K_2 were calculated to be 150 (± 10) M^{-1} and 72 (± 7) M^{-1}, respectively.

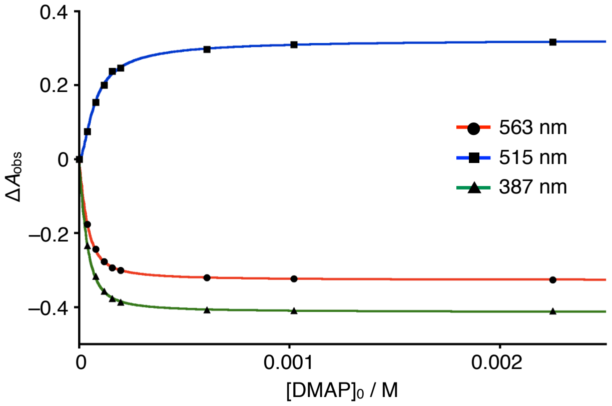

Figure S12. UV-visible absorption titration plot of **1a** with DMAP. The absorbance at 563, 515 and 387 nm of a toluene solution of **1a** (1.2×10^{-5} M) were monitored (black markers). The fitting curves were delineated for the determination of the binding constants of **1a** toward DMAP (lines). K_1 and K_2 were calculated to be 3.8 (± 0.3) × 10^4 M^{-1} and 2.3 (± 0.1) × 10^4 M^{-1}, respectively.

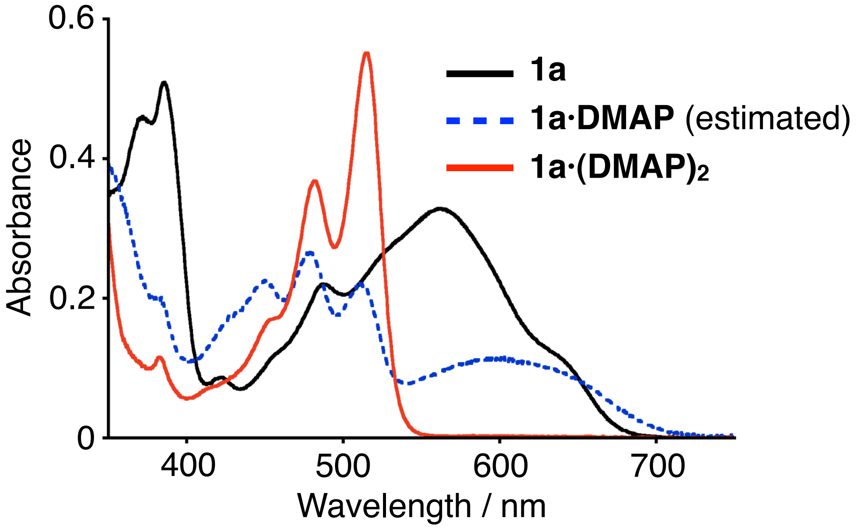

Figure S13. Absorption spectra of **1a** and **1a·(DMAP)$_2$** in THF and an estimated spectrum of , **1a·DMAP**: The spectrum of **1a·DMAP** was obtained by the nonlinear least-square fitting analysis of the titration spectra. The onset of the absorption spectrum of **1a·DMAP** was observed at longer wavelength, when compared with those of **1a** and **1a·(DMAP)$_2$**.

5. Electrochemical reduction of 1a

Electrochemical reduction of **1a** was performed using an Als/chi-617A electrochemical analyzer in the thin quarts cell (1 mm) equipped with a Pt mesh working electrode, a Pt wire counter electrode, and a $Ag/AgNO_3$ reference electrode. Tetrabutylammonium hexafluorophosphate ($n Bu_4NPF_6$) was used as a supporting electrolyte (0.1 M). While applying a voltage, spectral changes in UV-visible absorption were periodically recorded on an Ocean Optics HR4000CG-UV-NIR high-resolution spectrometer with a DH-2000-BAL UV-visible-NIR light source. The measurements were carried out under an argon atmosphere.

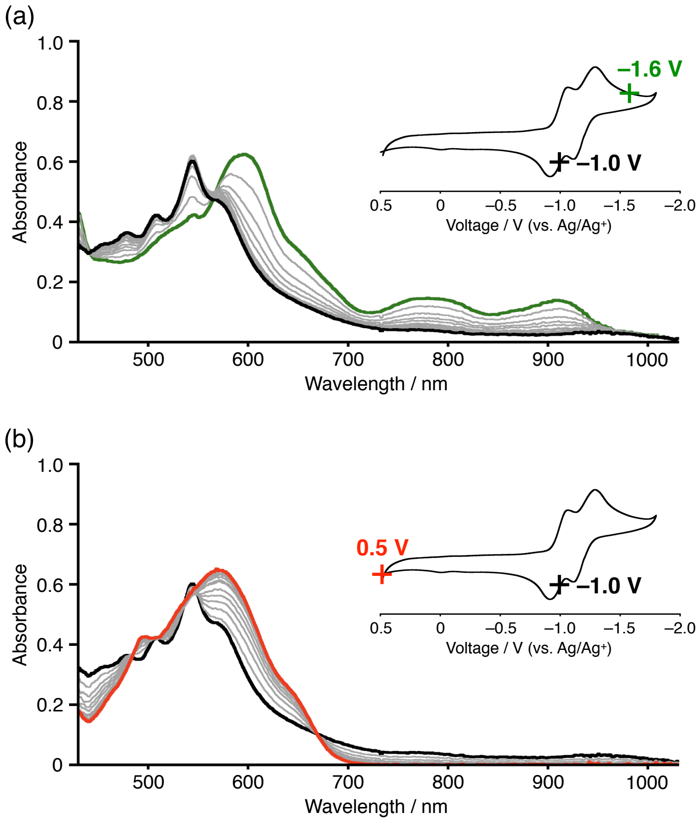

Figure S14. Changes in absorption spectra of **1a** in *o*-dichlorobenzene (3.4×10^{-4} M) on the reverse process of the electrochemical reduction: (a) $E_{app} = -1.0$ V and (b) $E_{app} = 0.5$ V.

6. Chemical reduction

Scheme S3.

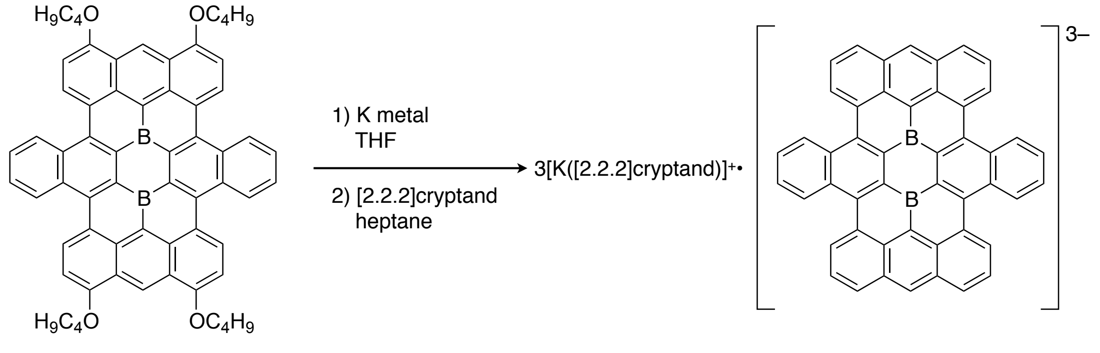

1b **3[K([2.2.2]cryptand)]$^{+}$·1^{3-}**

Chemical reduction of 1b with potassium metal. To a solution of **1b** (1.8 mg, 2.0 μmol) in THF (2 mL) was added freshly cut potassium metal (0.9 mg, 23 μmol). The resulting mixture was stirred for 2 days at room temperature. The dark color solution was layered with a solution of [2.2.2]cryptand in heptane to afford black crystals of an over-reduced species **3[K([2.2.2]cryptand)]$^{+}$·1^{3-}**. The structure was determined by X-ray crystallographic analysis (Figure S14).

Scheme S4.

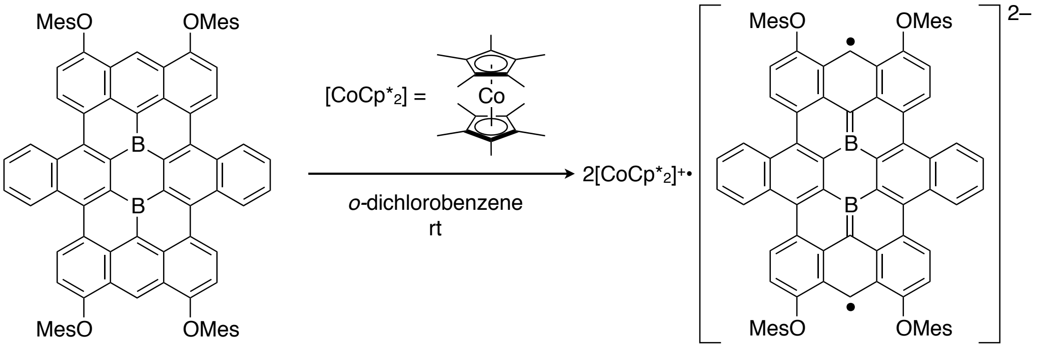

1a **2[CoCp*$_2$]$^{+}$·1a^{2-}**

Chemical reduction of 1a with [CoCp*$_2$]. To a solution of **1a** (1.6 mg, 1.3 μmol) in *o*-dichlorobenzene (4 mL) was added [CoCp*$_2$] (1.3 mg, 3.4 μmol). The mixture was stirred for 5 min at room temperature to yield a deep green solution of **2[CoCp*$_2$]$^{+}$·1a^{2-}**. The UV-visible

absorption spectrum, which showed absorption maxima around 600, 780, 890 nm, was consistent with that obtained by electrochemical reduction.

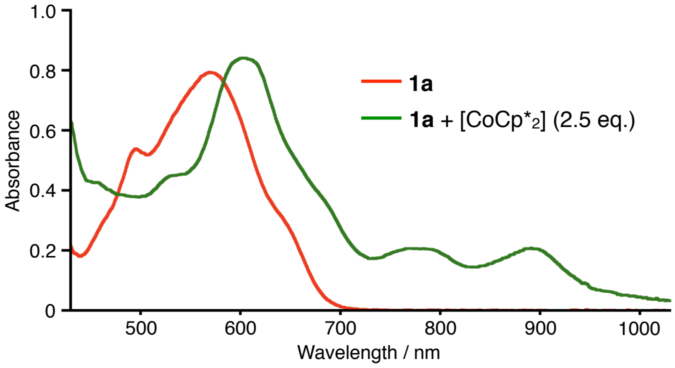

Figure S15. UV-visible-NIR absorption spectra of **1a** before (red line) and after (green line) addition of [CoCp*$_2$] (2.5 eq.).

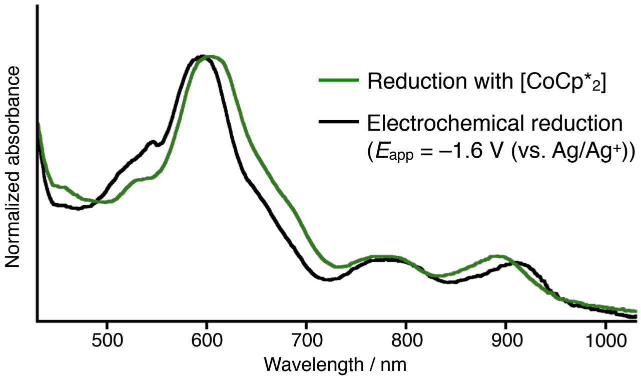

Figure S16. Comparison of UV-visible-NIR absorption spectra of **1a**$^{2-}$ generated by chemical reduction (green line) and electrochemical reduction (black line).

7. Variable-temperature ESR analysis of $2[CoCp^*_2]^+ \cdot$**1a**$^{2-}$

ESR spectra were recorded with a JEOL JES-FA 200 ESR spectrometer in the temperature range of 4–200 K. The ESR sample was prepared as follows: To a solution of **1a** (2.3 mg, 2.0 μmol) in *o*-dichlorobenzene (6 mL) was added $[CoCp^*_2]$ (2.0 mg, 6.0 μmol). The reaction mixture was stirred for 5 min and transferred to an ESR tube equipped with a two-way cock. The tube was flame-sealed under high vacuum while freezing the sample solution with liquid nitrogen.

The energy gap between open singlet state and triplet state (ΔE_{ST}) was estimated by curve-fitting of I (intensity) × T (temperature) plot with the following equation (Bleaney-Bowers equation):

$$I = \frac{3e^{-\Delta E_{ST}/RT}}{1+3e^{-\Delta E_{ST}/RT}} \frac{C}{T}$$

8. Cyclic voltammetry of 1c

Cyclic voltammetry (CV) was performed on an Als/chi-617A electrochemical analyzer. The CV cell consisted of a glassy carbon working electrode, a Pt wire counter electrode, and a Ag/$AgNO_3$ reference electrode. The measurements were carried out under an argon atmosphere at a scan rate of 50 mV/s with *n*Bu_4NPF_6 as a supporting electrolyte (0.1 M). The redox potentials were calibrated with ferrocene as an internal standard.

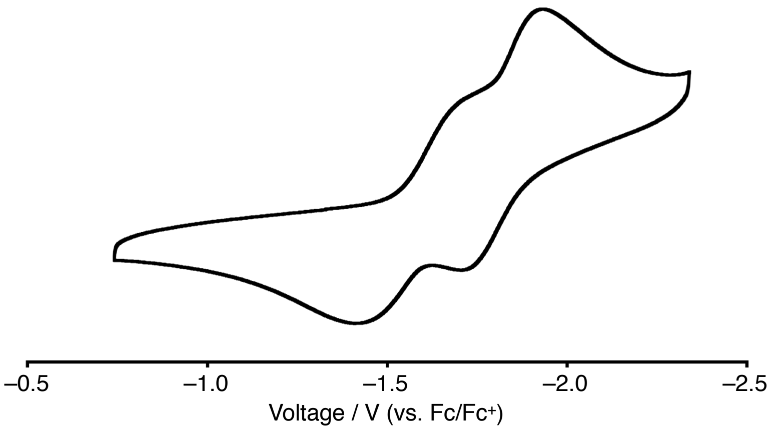

Figure S17. Cyclic voltammogram of **1c** in *o*-dichlorobenzene.

9. Evaluation of battery electrode performance

The charge/discharge tests were performed with a Hokuto HJ1001-SM8A charge/discharge device. Specific capacity is determined based on the weight of active materials. The theoretical capacity *C* was calculated from the following equation:[S5]

$$C(mAhg^{-1}) = \frac{N_A \cdot e}{3600 \cdot (M_W / 1000)}$$

where $N_A{\cdot}e$ is the Faraday constant (96487 C mol^{-1}), and M_w is the molecular weight required to store one electron.

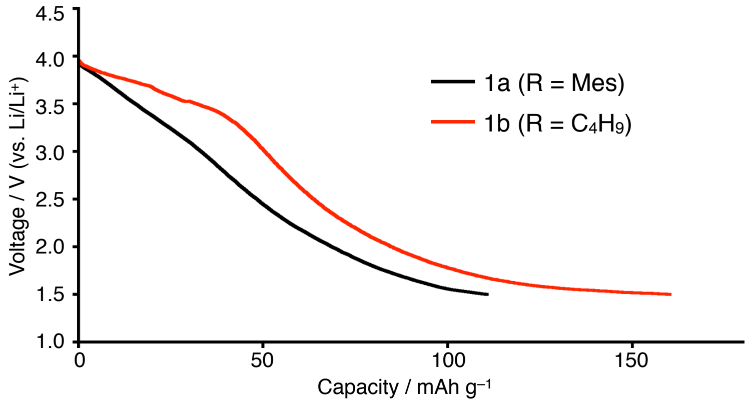

Figure S18. The first discharge profile of Li batteries with the working electrode composed of 10% **1a** (black) or **1b** (red) as active materials, 70% carbon black as a conductive agent, and 20 wt% polyvinylidene difluoride as a binder. Constant current density: 0.5 mA cm^{-2}. Voltage range: 1.5–4.0 V.

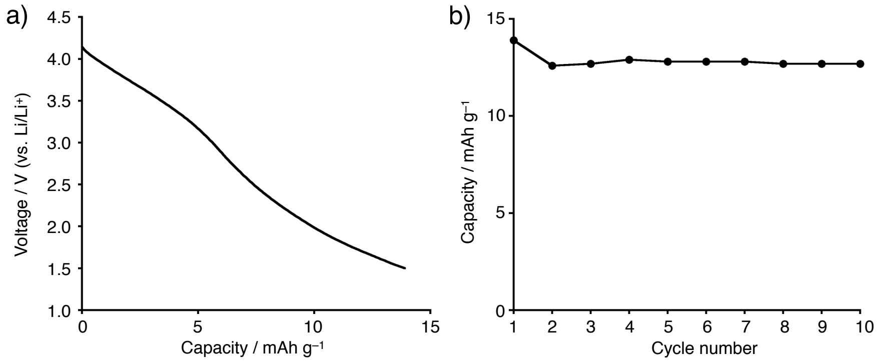

Figure S19. (a) The first discharge profile and (b) cycle performance of a reference Li battery with the working electrode composed of 80 wt% carbon black and 20 wt% polyvinylidene difluoride. Constant current density: 0.5 mA cm^{-2}. Voltage range: 1.5–4.1 V.

10. X-ray crystallographic analysis

1b. Single crystals of **1b** suitable for X-ray crystallographic analysis were obtained by slow diffusion of 2-propanol into a solution of **1b** in 1,2-dichloroethane. The intensity data were collected at 103 K with a Rigaku X-ray diffractometer equipped with a molybdenum MicroMax-007 microfocus generator, VariMax-Mo optics, and a Saturn 70 CCD detector. Total 7610 reflections were collected, among which 3847 reflections were independent (R_{int} = 0.0877). The structure was solved by direct methods (SHELXS-2013) and refined by full-matrix least-squares on F^2 for all reflections (SHELXL-2013).[S6] All non-hydrogen atoms were refined anisotropically and all hydrogen atoms were placed using AFIX instructions. The crystal data are as follows: formula $C_{64}H_{54}B_2O_4$; FW = 908.69, triclinic, crystal size 0.05 × 0.01 × 0.01 mm, *P*-1, a = 7.553(5) Å, b = 12.357(7) Å, c = 12.549(8) Å, α = 83.18(4)°, β = 74.39(2)°, γ = 84.50(4)°, V = 1117.6(12) Å^3, Z = 1, D_{calcd} = 1.350 g cm^{-3}, R_1 = 0.0694 ($I > 2s(I)$), wR_2 = 0.1597 (all data), GOF = 0.947. CCDC registration number 1059942.

1c. Single crystals of **1c** were obtained by slow diffusion of octane into a solution of **1c** in chlorobenzene. The intensity data were collected at 103 K with a Rigaku X-ray diffractometer equipped with a molybdenum MicroMax-007 microfocus generator, VariMax-Mo optics, and a Saturn 70 CCD detector. Total 34013 reflections were collected, among which 13230 reflections were independent (R_{int} = 0.0818). The structure was solved by direct methods (SHELXD-2013) and refined by full-matrix least-squares on F^2 for all reflections (SHELXL-2013).[S6] All non-hydrogen atoms were refined anisotropically and all hydrogen atoms were placed using AFIX instructions. The crystal data are as follows: formula $C_{96}H_{118}B_2O_4$; FW = 1357.52, triclinic, *P*-1, a = 11.769(12) Å, b = 14.134(13) Å, c = 23.62(2) Å, α = 84.13(3)°, β = 87.54(2)°, γ = 84.78(2)°, V = 3890(7) Å^3, Z = 2, D_{calcd} = 1.190 g cm^{-3}, R_1 = 0.1016 ($I > 2s(I)$), wR_2 = 0.3144 (all data), GOF = 1.064. CCDC registration number 1059943.

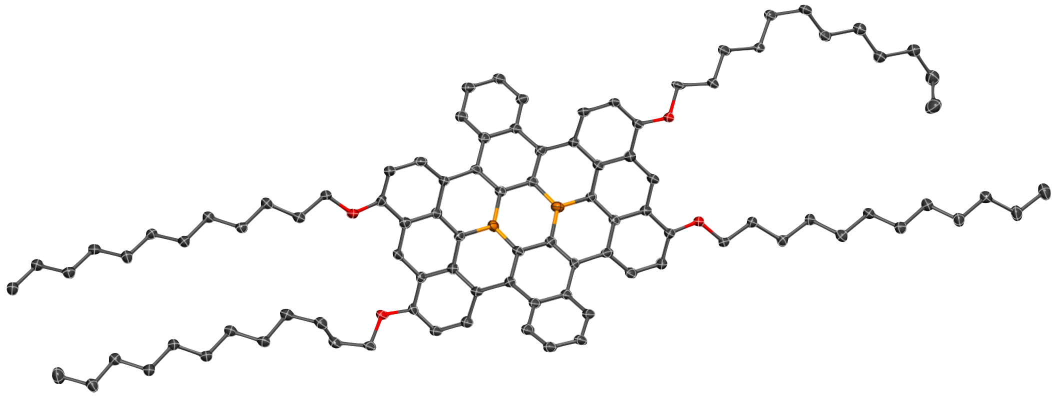

Figure S20. X-ray crystal structure of **1c**. The thermal ellipsoids are scaled to the 50% probability level. Hydrogen atoms were omitted for clarity. Orange: boron. Red: oxygen.

$3[K([2.2.2]cryptand)]^{+}\cdot 1^{3-}$. Single crystals were obtained by slow diffusion of a [2.2.2]cryptand heptane solution into the reaction mixture of **1b** and potassium metal. The intensity data were collected at 103 K with a Rigaku X-ray diffractometer equipped with a molybdenum MicroMax-007 microfocus generator, VariMax-Mo optics, and a Saturn 70 CCD detector. Total 85075 reflections were collected, among which 16578 reflections were independent (R_{int} = 0.0628). The structure was solved by direct methods (SHELXS-2013) and refined by full-matrix least-squares on F^2 for all reflections (SHLEXL-2013).[S6] All non-hydrogen atoms were refined anisotropically and all hydrogen atoms were placed using AFIX instructions. The crystal data are as follows: Formula $C_{102}H_{130}B_2K_3N_6O_{18}$; FW = 1867.03, monoclinic, crystal size 0.15 × 0.10 × 0.05 mm, $P2_1/n$, a = 15.4324(12) Å, b = 29.3994(18) Å, c = 21.2075(13) Å, β = 97.632(2)°, V = 9536.7(11) Å^3, Z = 4, D_{calcd} = 1.300 g cm^{-3}; R_1 = 0.0880 (I > 2s(I)), wR_2 = 0.2351 (all data), GOF = 1.150. CCDC registration number 1059944.

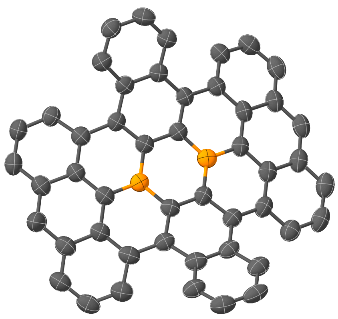

Figure S21. X-ray crystal structure of **3[K([2.2.2]cryptand)]$^+$·1^{3-}**. The thermal ellipsoids are scaled to the 50% probability level. Hydrogen atoms and counter cations were omitted for clarity. Orange: boron.

1b·(MePy)$_2$. Single crystals of **1b·(MePy)$_2$** were obtained by slow diffusion of heptane into a solution of **1b** in 4-methylpyridine. The intensity data were collected at 123 K with a Rigaku X-ray diffractometer equipped with a copper MicroMax-007 microfocus generator, VariMax-Cu optics, and a RAPID IP detector. Total 74212 reflections were collected, among which 12594 reflections were independent (R_{int} = 0.0460). The structure was solved by direct methods (SHELXS-2013) and refined by full-matrix least-squares on F^2 for all reflectioins (SHELXL-2013).[S6] All non-hydrogen atoms were refined anisotropically and all hydrogen atoms were placed using AFIX instructions. The crystal data are as follows: Formula $C_{88}H_{82}B_2N_4O_4$; FW = 1281.19, monoclinic, crystal size 0.42 × 0.17 × 0.01 mm, $P2_1/c$, a = 17.0469(3) Å, b = 17.6986(3) Å, c = 23.1757(4) Å, β = 99.4921(9)°, V = 6896.5(2) Å^3, Z = 4, D_{calcd} = 1.234 g cm^{-3}; R_1 = 0.0555 (I > 2s(I)), wR_2 = 0.1574 (all data), GOF = 1.063. CCDC registration number 1059945.

11. Theoretical calculations

Structural optimizations of 1a and 1b. Structural optimizations were performed using Gaussian 09 program[S7] at the B3LYP/6-31G* level of theory.

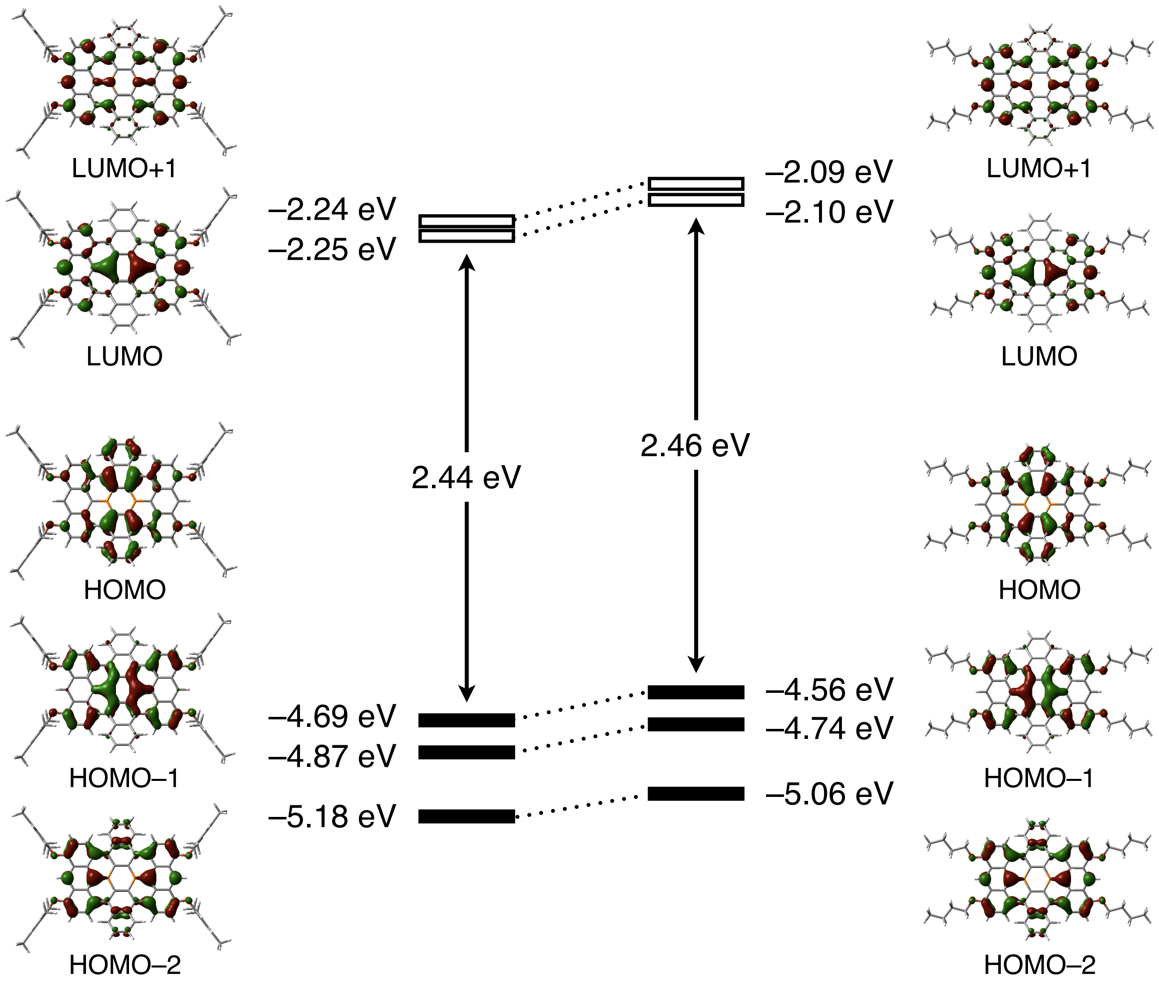

Figure S22. Comparison of the electronic structures between **1a** (left) and **1b** (right).

Structural optimization of 1b′·(MePy)₂. Structural optimizations of *cis* and *trans* complexes of **1b′** with 4-methylpyridine, where the peripheral substituents were replaced by methoxy groups for simplicity, were performed using Gaussian 09 program[S7] at the B3LYP/6-311G** level of theory.

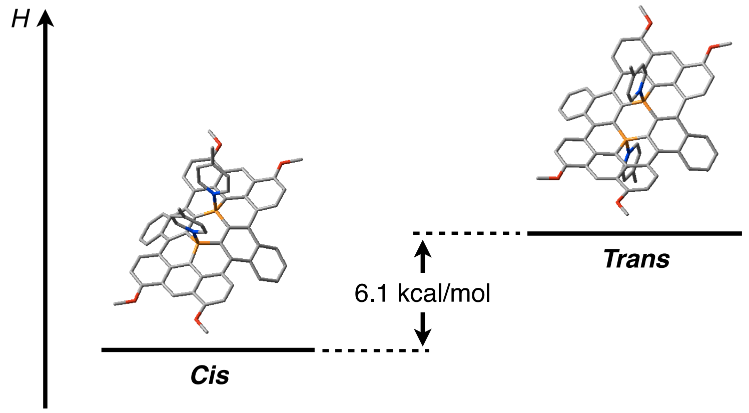

Figure S23. Optimized structures and relative energies of *cis*- and *trans*-isomers of **1′·(MePy)₂**. Hydrogen atoms were omitted for clarity.

Structural optimization and TD-DFT calculations of 1b′, 1b′·DMAP, and 1b′·(DMAP)$_2$. Structures of **1b′** and its Lewis adducts with DMAP, **1b′·DMAP** and **1b′·(DMAP)$_2$**, were optimized and then TD-DFT calculations were performed at the B3LYP/6-31G* level of theory. The mono Lewis adduct **1b′·DMAP** has a donor-acceptor type electronic structure, in which the HOMO is delocalized around the tetra-coordinate boron moiety while the LUMO around the tri-coordinate boron unit. Therefore, the $S_0 \rightarrow S_1$ transition energy of **1b′·DMAP** is smaller than those of **1b′** and **1b′·(DMAP)$_2$**.

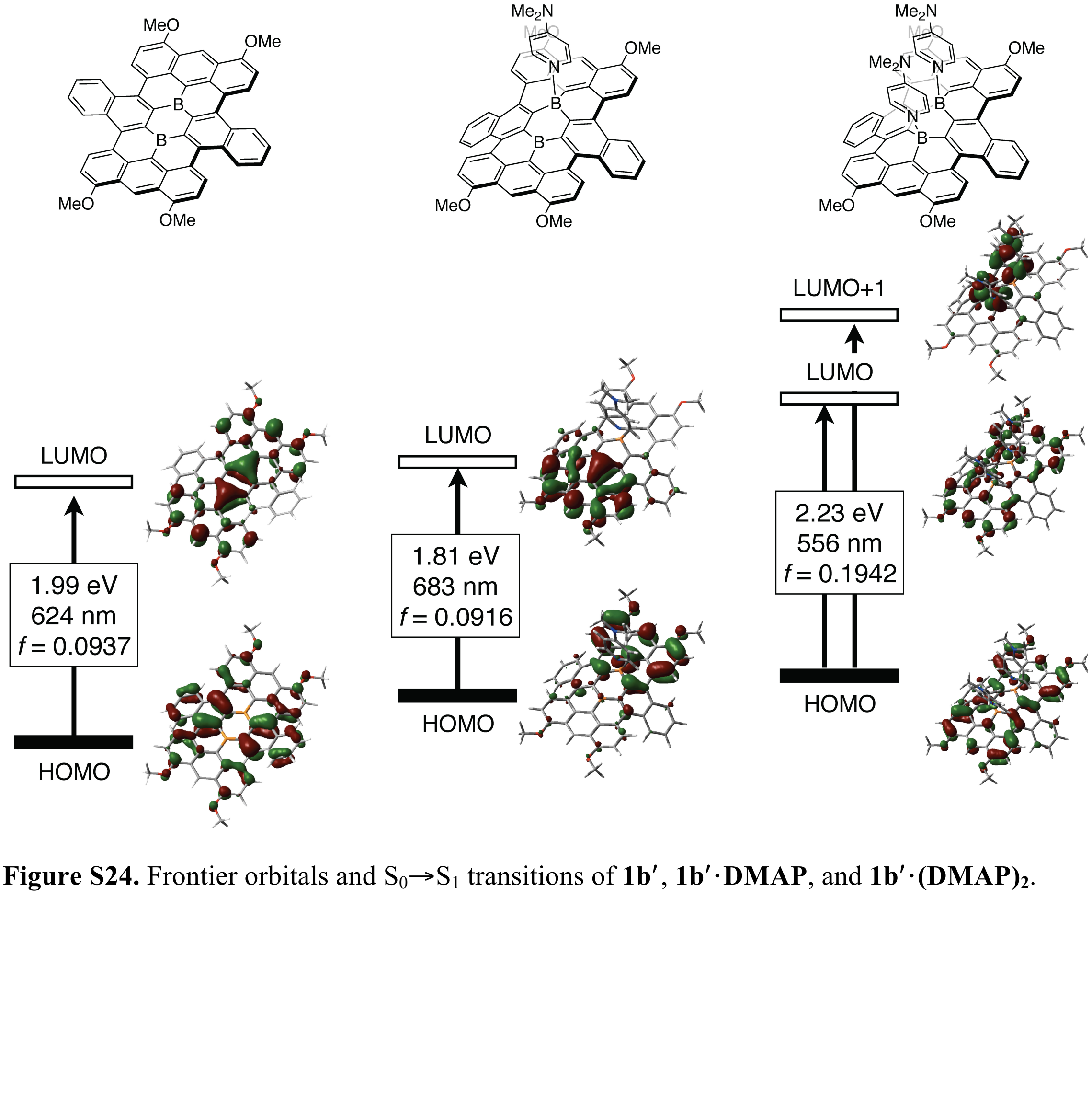

Figure S24. Frontier orbitals and $S_0 \rightarrow S_1$ transitions of **1b′**, **1b′·DMAP**, and **1b′·(DMAP)$_2$**.

Structural optimization of 1b′$^{2-}$. Structural optimizations of **1b′**$^{2-}$ were performed using Gaussian 09 program[S7] at the (U)B3LYP/6-311+G** level of theory. The symmetry-broken approach was applied for open-shell singlet calculations. Singlet biradical character (y) was calculated by the natural orbital occupancy number (NOON) of the LUMO in the broken-symmetry UHF/6-311+G** calculation[S8] using the Yamaguchi scheme[S9].

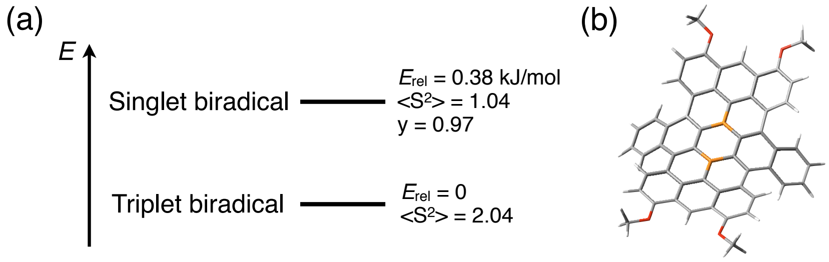

Figure S25. (a) Relative energies of **1b′**$^{2-}$ in different spin states and (b) optimized structure of the triplet biradical state. Orange: boron. Red: oxygen.

12. References

(S1) N. K. S. Davis, M. Pawlicki and H. L. Anderson, *Org. Lett.* 2008, **10**, 3945.

(S2) J. Chen, J. W. Kampf and A. J. Ashe III, *Organometallics* 2008, **27**, 3639.

(S3) S. L. S Jacoby, J. S. Kowalik and J. T. Pizzo, *Interative Methods for Nonlinear Optimization Problems*, Prentice Hall, Inc., New Jersey, 1972.

(S4) Y. Kuroda, http://www.eonet.ne.jp/~spana-lsq/index.html.

(S5) J. Qu, T. Katsumata, M. Satoh, J. Wada, J. Igarashi, K. Mizoguchi and T. Masuda, *Chem. Eur. J.* 2007, **13**, 7965.

(S6) T. Gruene, H. W. Hahn, A. V. Luebben, F. Meilleurb and G. M. Sheldrick, *J. Appl. Cryst.* 2014, **47**, 462.

(S7) Gaussian 09 (Revision C.01), M. J. Frisch, G. W. Trucks, H. B. Schlegel, G. E. Scuseria, M. A. Robb, J. R. Cheeseman, G. Scalmani, V. Barone, B. Mennucci, G. A. Petersson, H. Nakatsuji, M. Caricato, X. Li, H. P. Hratchian, A. F. Izmaylov, J. Bloino, G. Zheng, J. L. Sonnenberg, M. Hada, M. Ehara, K. Toyota, R. Fukuda, J. Hasegawa, M. Ishida, T. Nakajima, Y. Honda, O. Kitao, H. Nakai, T. Vreven, J. A. Montgomery, Jr., J. E. Peralta, F. Ogliaro, M. Bearpark, J. J. Heyd, E. Brothers, K. N. Kudin, V. N. Staroverov, R. Kobayashi, J. Normand, K. Raghavachari, A. Rendell, J. C. Burant, S. S. Iyengar, J. Tomasi, M. Cossi, N. Rega, J. M. Millam, M. Klene, J. E. Knox, J. B. Cross, V. Bakken, C. Adamo, J. Jaramillo, R. Gomperts, R. E. Stratmann, O. Yazyev, A. J. Austin, R. Cammi, C. Pomelli, J. W. Ochterski, R. L. Martin, K. Morokuma, V. G. Zakrzewski, G. A. Voth, P. Salvador, J. J. Dannenberg, S. Dapprich, A. D. Daniels, O. Farkas, J. B. Foresman, J. V. Ortiz, J. Cioslowski and D. J. Fox, Gaussian, Inc., Wallingford CT, 2010.

(S8) (a) D. Döhnert and J. Koutecký, *J. Am. Chem. Soc.* 1980, **102,** 1789. (b) Y. Jung and M. Head-Gordon, *ChemPhysChem*, 2003, **4**, 522.

(S9) K. Yamaguchi, *Chem. Phys. Lett.* 1975, **33**, 330.

13. NMR spectra

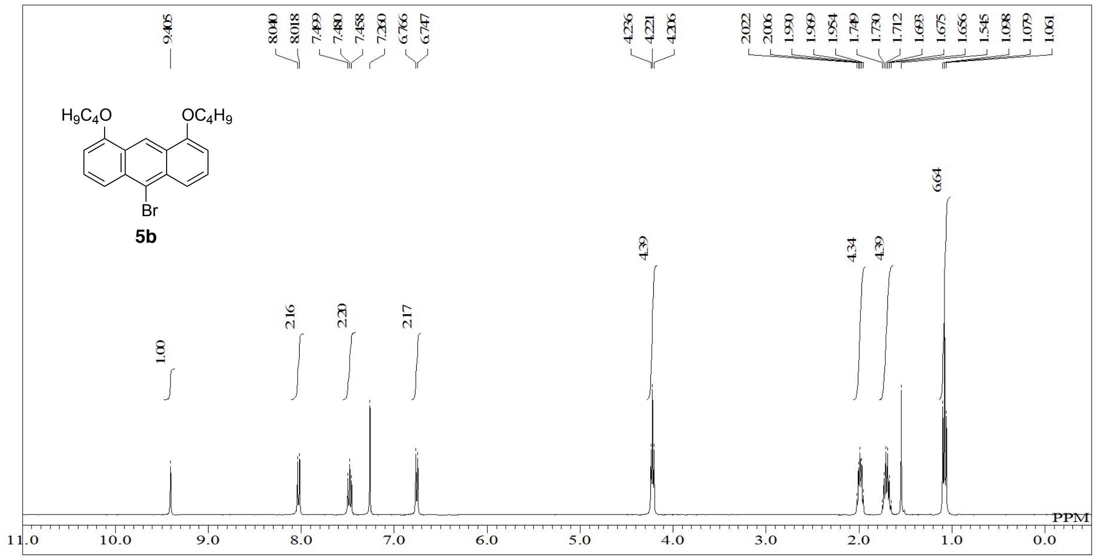

Figure S26. ^{1}H NMR spectrum (400 MHz) of **5b** in $CDCl_3$.

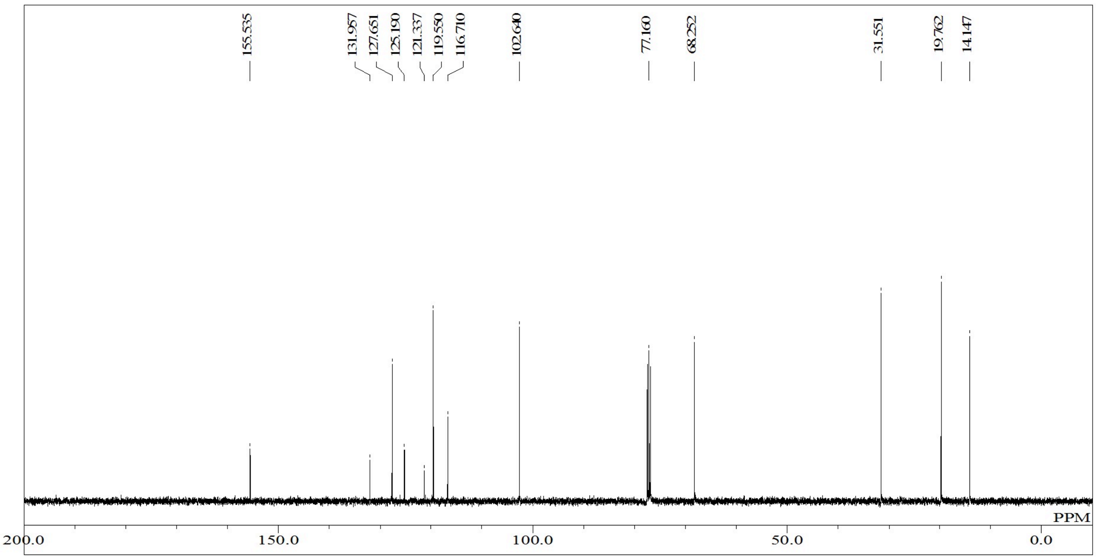

Figure S27. ^{13}C NMR spectrum (100 MHz) of **5b** in $CDCl_3$.

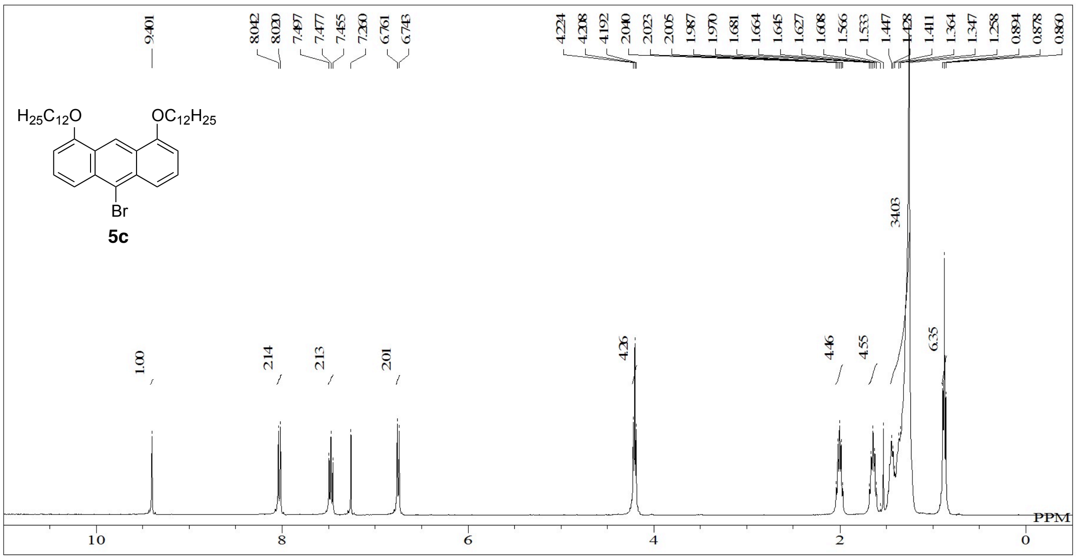

Figure S28. ^{1}H NMR spectrum (400 MHz) of **5c** in $CDCl_3$.

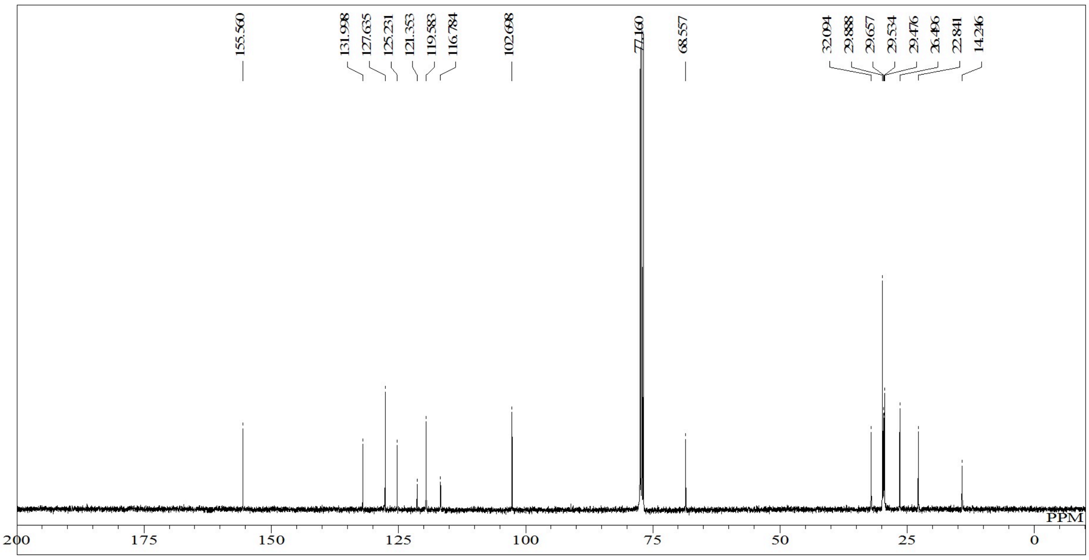

Figure S29. ^{13}C NMR spectrum (100 MHz) of **5b** in $CDCl_3$.

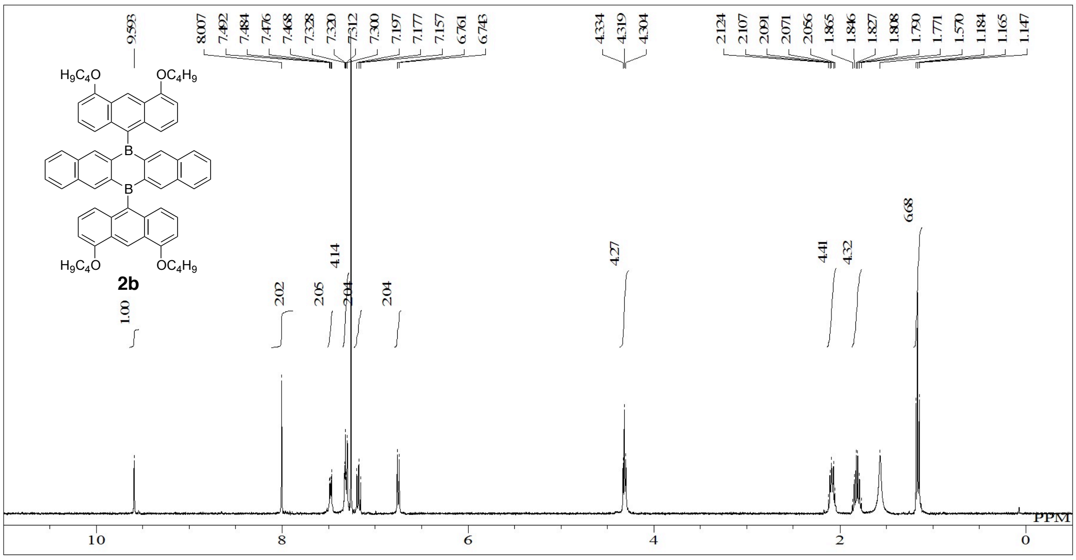

Figure S30. ^{1}H NMR spectrum (400 MHz) of **2b** in $CDCl_3$.

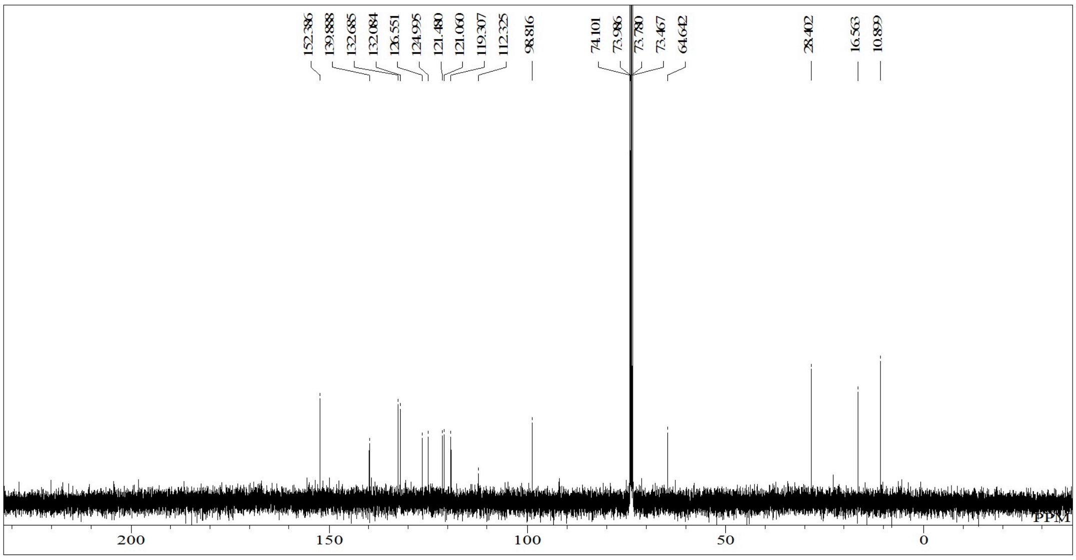

Figure S31. ^{13}C NMR spectrum (100 MHz) of **2b** in $C_2D_2Cl_4$.

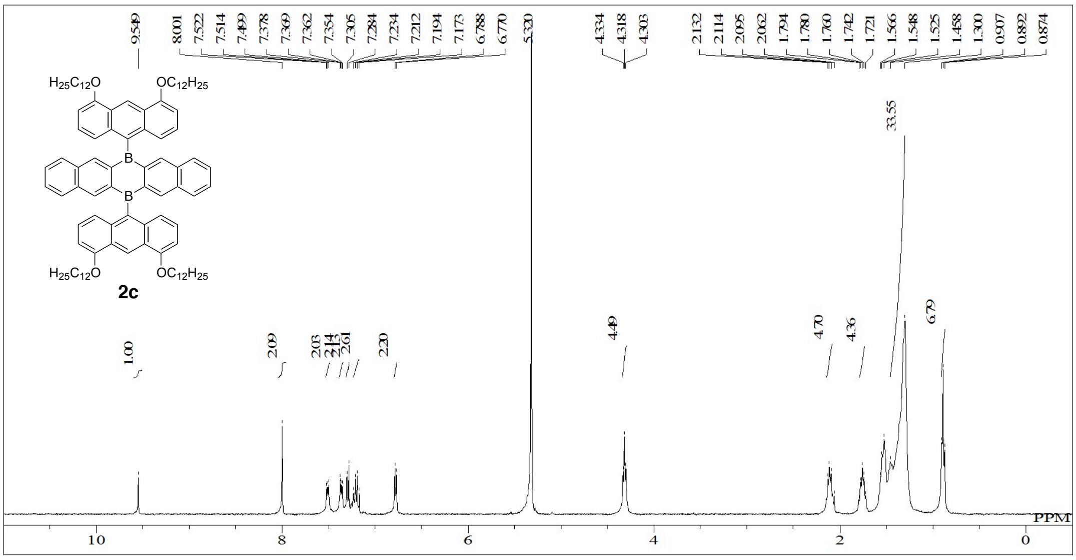

Figure S32. ^{1}H NMR spectrum (400 MHz) of **2c** in CD_2Cl_2.

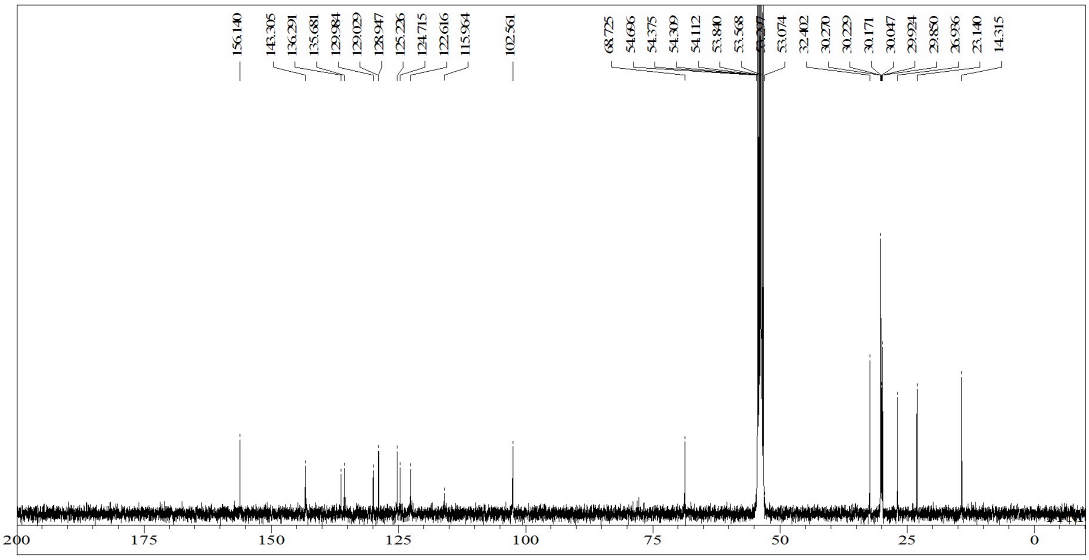

Figure S33. ^{13}C NMR spectrum (100 MHz) of **2b** in CD_2Cl_2.

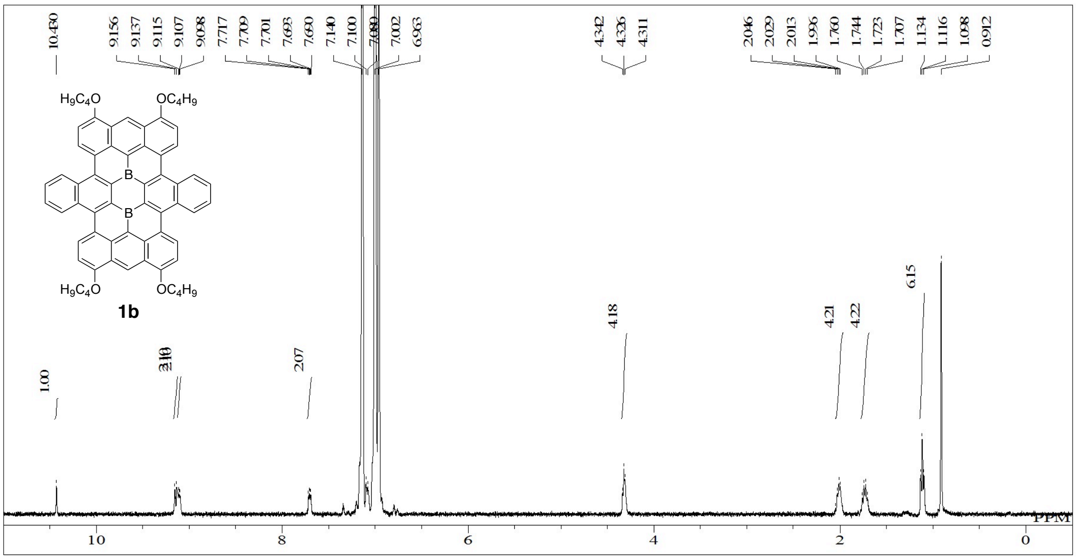

Figure S34. ^{1}H NMR spectrum (400 MHz) of **1b** in C_6D_5Cl at 110 °C.

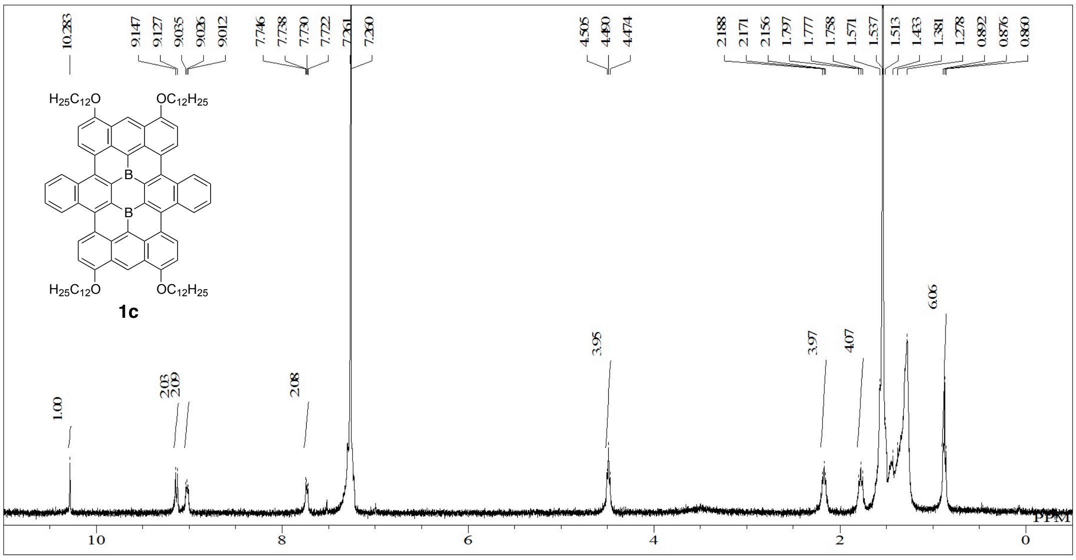

Figure S35. ^{1}H NMR spectrum (400 MHz) of **1c** in $CDCl_3$.

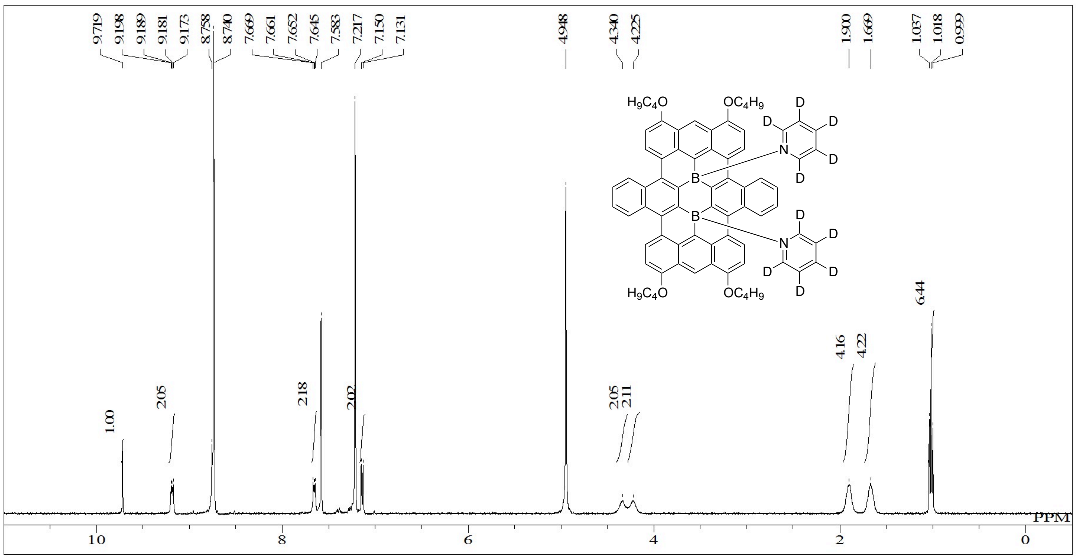

Figure S36. ^{1}H NMR spectrum (400 MHz) of **1b** in C_5D_5N.

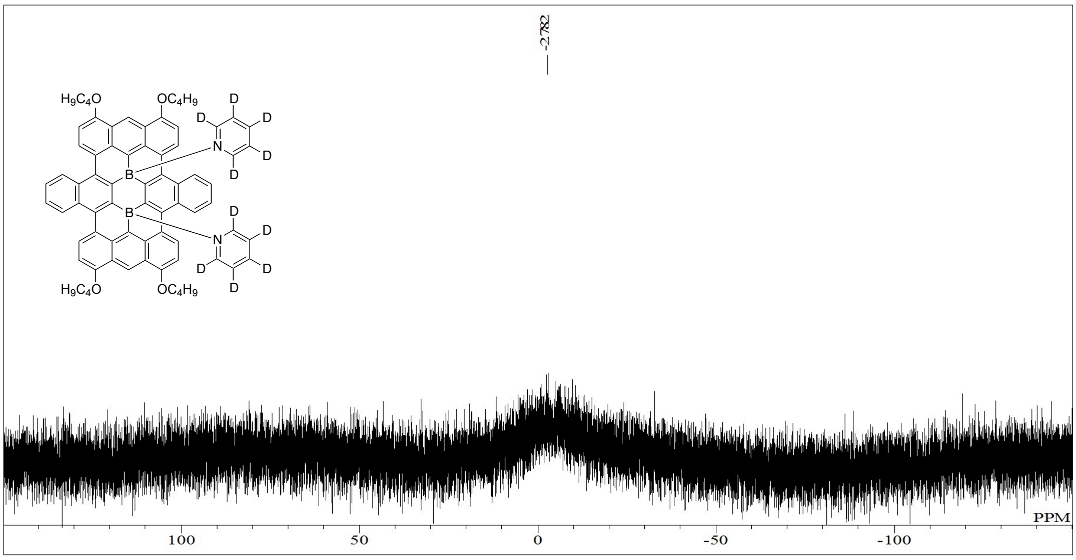

Figure S37. ^{11}B NMR spectrum (128 MHz) of **1b** in C_5D_5N.
